# Covalent Adaptable Polymethacrylate Networks by Hydrazide Crosslinking Via Isosorbide Levulinate Side Groups

**DOI:** 10.1021/acssuschemeng.3c00747

**Published:** 2023-05-19

**Authors:** Livia Matt, Rauno Sedrik, Olivier Bonjour, Miglé Vasiliauskaité, Patric Jannasch, Lauri Vares

**Affiliations:** † Institute of Technology, University of Tartu, Nooruse 1, Tartu 50411, Estonia; ‡ Department of Chemistry, Lund University, P.O. Box 124, Lund 221 00, Sweden

**Keywords:** isosorbide, polymethacrylate, vinyl levulinate, *Candida antarctica* lipase B, adipic dihydrazide, dynamic covalent bond, covalent adaptable network, biobased polymer

## Abstract

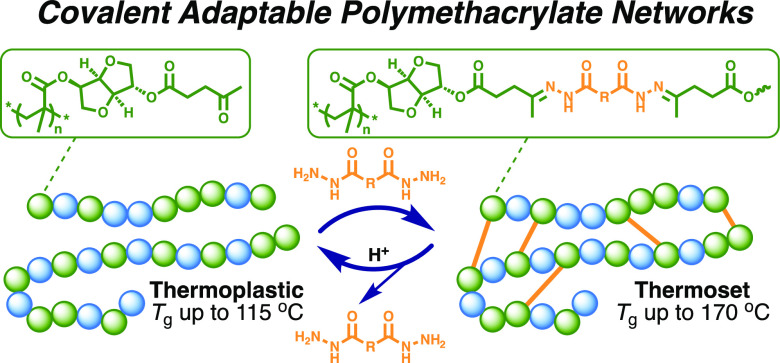

Reversible crosslinking offers an attractive strategy to modify and improve the properties of polymer materials while concurrently enabling a pathway for chemical recycling. This can, for example, be achieved by incorporating a ketone functionality into the polymer structure to enable post-polymerization crosslinking with dihydrazides. The resulting covalent adaptable network contains acylhydrazone bonds cleavable under acidic conditions, thereby providing reversibility. In the present work, we regioselectively prepare a novel isosorbide monomethacrylate with a pendant levulinoyl group via a two-step biocatalytic synthesis. Subsequently, a series of copolymers with different contents of the levulinic isosorbide monomer and methyl methacrylate are prepared by radical polymerization. Using dihydrazides, these linear copolymers are then crosslinked via reaction with the ketone groups in the levulinic side chains. Compared to the linear prepolymers, the crosslinked networks exhibit enhanced glass transition temperatures and thermal stability, up to 170 and 286 °C, respectively. Moreover, the dynamic covalent acylhydrazone bonds are efficiently and selectively cleaved under acidic conditions to retrieve the linear polymethacrylates. We next show that recovered polymers can again be crosslinked with adipic dihydrazide, thus demonstrating the circularity of the materials. Consequently, we envision that these novel levulinic isosorbide-based dynamic polymethacrylate networks have great potential in the field of recyclable and reusable biobased thermoset polymers.

## Abstract 2

Reversible polymethacrylate networks have been developed from biobased isosorbide and levulinic acid.

## Introduction

Rapidly increasing environmental concerns and tighter governmental regulations drive the scientific community and industry to focus on renewable feedstocks such as lignocellulosic biomass to produce chemicals and materials for our everyday life.^1^ Currently, most plastics are still produced from fossil feedstock,^2^ and it is challenging to develop biobased plastics that are easily processable, economically viable to manufacture, and at the same time have all the desired physicochemical properties needed for various applications.^3^ Preparation of biobased thermoplastics with high glass transition temperatures (*T*_g_ > 100 °C) is particularly demanding since the availability of suitable rigid building blocks from biomass, required to achieve high-*T*_g_ polymers, is limited.^4^ In this context, isosorbide,^5^ a stiff bicyclic diol produced from d-glucose on an industrial scale,^6^ has great potential for replacing its fossil-based counterparts in high-performance plastics in a cost-competitive way.^7,8^ Because of the ease of preparation and the structural rigidity, this V-shaped secondary diol^9^ has been extensively studied in various step-growth polymers.^7,10^ Some isosorbide-based polyesters and polycarbonates have also reached the market.^11,12^ We and others have recently developed regioselective functionalization methods of the chemically non-equivalent *endo*- and *exo*-OH groups of isosorbide. This has opened a path toward the synthesis of thermoplastic chain-growth polymers such as poly(meth)acrylates^13−17^ and polyethers^18,19^ with rigid isosorbide units as pendant groups.

Another strategy to enhance and vary the properties (e.g., increase hardness, *T*_g_, thermal stability, and solvent resistance) with the aim of widening the applicability is to transform a thermoplastic polymer into a thermoset by connecting the linear polymer chains via dynamic multifunctional crosslinkers.^20^ Such dynamic covalent polymer networks,^21^ also known as covalent adaptable networks (CANs),^22^ may provide chemical stability and mechanical strength equal to that provided by conventional non-dynamic crosslinked polymer networks.^23^ CANs have also been studied from different biobased sources, e.g., vegetable oils, lignin, and sugars.^24^ Dynamic covalent chemistries studied include, e.g., transesterifications,^25,26^ disulfide metathesis,^27^ Diels–Alder reaction,^28^ carbamate exchange,^29^ dynamic imine,^30^ acetal,^31^ urea,^32^ and boronic ester bonds,^33^ and silyl ether exchange reactions.^34^ Recyclable isosorbide-based polybenzoxazine vitrimers with self-healing capabilities have been reported by Verge.^35^ Under specific circumstances (e.g., in acidic/basic environment, UV irradiation, temperature change, etc.), these dynamic crosslinks can be cleaved to recover the linear thermoplastic polymers.^36^ Thus, this strategy is advantageous because of the recyclability and reusability of polymeric materials.^37,38^ In this approach, a suitable chemical functionality, which enables post-polymerization reaction with a crosslinking agent, should be incorporated into the monomer structure. For instance, the incorporation of a ketone or aldehyde functionality in the polymer structure allows the introduction of a reversible crosslinking feature. For example, the reaction of a carbonyl group in an aldehyde or a ketone with a hydrazide group results in an acylhydrazone bond, which can be hydrolyzed under acidic conditions.^37,38^ Consequently, the acylhydrazone bond is a dynamic covalent bond that can be reversibly cleaved.

Adipic dihydrazide (**adh**) has been studied as a crosslinking agent capable of forming acylhydrazone bonds with aldehydes and ketones.^39−45^ This C6-dihydrazide is typically prepared from diethyl ester of adipic acid and hydrazine hydrate,^46,47^ and is considered safe in food contact materials^48^ and is thus widely used in industry.^49^ Levulinic acid, commercially produced from lignocellulosic feedstock,^50,51^ is an appealing building block with ketone and carboxylic acid functionalities. These functionalities enable levulinic acid to be converted into other chemicals relevant for various applications.^52,53^ Furthermore, levulinic acid is considered to be one of the most promising bioderived building blocks by the US Department of Energy.^54^

Acylhydrazone bonds formed through the reaction of a levulinic ketone group and a hydrazide unit have previously been reported to produce compounds^55^ and drug delivery systems.^56−59^ In addition to derivatives with one levulinoyl group, compounds with two levulinic units can also be incorporated into a molecular structure through acylhydrazone bonds, e.g., in the main chain of polyacylhydrazones.^44^ Even though levulinic acid is a promising platform chemical for the production of polymers,^60^ there are, to the best of our knowledge, no previous examples reported where levulinic unit has been exploited in post-polymerization crosslinking with dihydrazides via dynamic acylhydrazone bonds.

**Figure 1:**
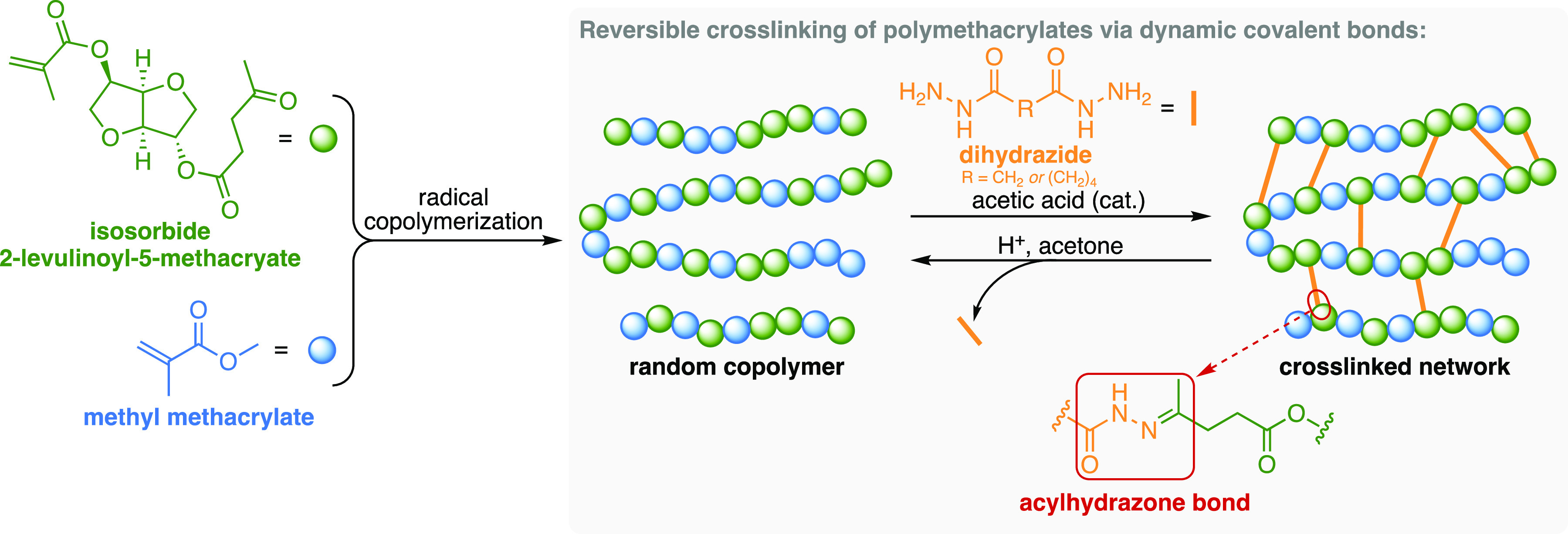
Simplified illustration of the reversible crosslinking of polymethacrylates via dynamic acylhydrazone bonds studied in the present work.

In the current work, we have regioselectively functionalized isosorbide with monomethacryl and levulinoyl side chains using readily upscalable enzymatic catalysis. Next, a series of copolymers based on the methacrylic isosorbide levulinate monomer and methyl methacrylate were prepared. These polymers enabled us to investigate the reversible crosslinking feature through the formation of dynamic covalent bonds between the pendant ketone groups in the levulinic side chain and different dihydrazides as crosslinking agents (Figure 1). Thereafter, the removal of the crosslinking unit under acidic conditions and a subsequent re-crosslinking with adipic dihydrazide were demonstrated. We hypothesize that this type of novel biobased covalent adaptable networks^24^ with a reversible crosslinking feature may be very useful in coating applications where curing of the material is needed. Moreover, the reversibility of the acylhydrazone bonds is highly favorable owing to the recyclability of this kind of thermoset materials.

## Experimental Section

### General

Isosorbide (98%) and levulinic acid (98%) were obtained from Acros Organics, vinyl methacrylate (98%) was obtained from Sigma-Aldrich, vinyl acetate (99%) was obtained from Fluka, adipic dihydrazide (**adh**, 97%) was obtained from Alfa Aesar, malonic dihydrazide (**mdh**, 99%) was obtained from Thermo Scientific, and methyl methacrylate (**MMA**, 99%) was obtained from Fisher Chemicals. All used solvents were HPLC grade. All reagents and solvents were used without further purification if not mentioned otherwise. Reactions were monitored by thin-layer chromatography (TLC), and TLC plates were visualized either by UV detection or by staining with phosphomolybdic acid (PMA) solution. The reaction products were purified by flash chromatography using silica gel 60 (0.040–0.063 mm, 230–400 mesh).

### Structural Characterization

The structures of the monomers and polymers were characterized by NMR spectroscopy using a Bruker 400 MHz spectrometer with the samples dissolved in chloroform-*d*. The ^1^H NMR and ^13^C NMR spectra were recorded at 400.1 and 100.6 MHz, respectively. The chemical shifts for the ^1^H and ^13^C NMR spectra are given in ppm and were calibrated using residual solvent signals (for ^1^H, CDCl_3_: δ = 7.26 ppm; for ^13^C, CDCl_3_: δ = 77.0 ppm). The following abbreviations are used for multiplicities: s, singlet; d, doublet; t, triplet; m, multiplet; and b, broadened. The formation of linear polymethacrylates [**PMMA**, **PIL**, **P(MMA**_***x***_**-IL**_***y***_**)**-s] from the corresponding monomethacrylates (**MMA**, **IL**) was determined by the decrease or disappearance of the proton signals of the double bond CH_2_=C between the 6.2 and 5.5 ppm region in comparison with the characteristic peaks of the polymers in ^1^H NMR spectra. The monomer ratios in copolymers were calculated from ^1^H NMR spectra by comparing the integrals of the **PMMA** signal at around 3.59 ppm (3H) and the **IL** signal at around 2.18 ppm (3H) or 4.47 ppm (1H) (for an example, see Figure S10). The **PMMA** tacticity was calculated based on the corresponding ^1^H NMR signals between 1.4 and 0.7 ppm (CH_3_–C, see Figure S4).^61^

For HRMS analysis of **IL**, a Varian FT-ICR-MS (with 7 T superconducting magnet) with a nanoESI ion source was used. An FTIR (ATR) spectrophotometer, Shimadzu IRAffinity-1, was used for IR analysis.

The molecular weights of the linear polymethacrylates and de-crosslinked polymers were determined by size-exclusion chromatography (SEC) in THF. The SEC setup included three Shodex columns coupled in series (KF-805, -804, and -802.5) situated in a Shimadzu CTO-20A prominence column oven, a Shimadzu RID-20A refractive index detector, and Shimadzu LabSolution software. All samples were run at 40 °C in THF at an elution rate of 1 mL min^–1^. Calibration was done using poly(methyl methacrylate) standards (*M*_n_ = 2990, 10,300, and 85,000 g mol^–1^).

### Thermal Characterization

Thermogravimetric analysis (TGA) was performed using TA Instruments TGA Q500 apparatus to determine the thermal stability of the polymers under a N_2_ atmosphere. The temperature was increased to 600 °C at a heating rate of 10 °C min^–1^. The thermal decomposition temperature (*T*_d,95_) was determined at 5% weight loss.

Differential scanning calorimetry (DSC) analysis was carried out using a TA Instruments DSC Q2000 differential scanning calorimeter. Dried samples were transferred to aluminum pans, which were hermetically sealed. The linear polymethacrylate and de-crosslinked polymer samples were first heated to 140–200 °C, then cooled to −50 °C, and finally heated to 140–200 °C. The (re-)crosslinked polymer samples were first heated to 160–200 °C, then cooled to −50 °C, and finally heated to 160–200 °C. The upper temperature limit depended on the *T*_d,95_ of the particular polymer under measurement. The scan rate in all cases was 10 °C min^–1^ during the temperature program. The *T*_g_ values of the polymers were evaluated from the second heating scans by identifying the inflection points.

### Solubility

The solubility of the polymers was investigated in a wide range of solvents, arranged according to their hydrogen-bonding capacity and solubility parameter (δ). Small samples (about 5 mg) of isosorbide-based polymers were mixed with a range of selected solvents (1 mL). Each mixture was stirred for 24 h at room temperature. The results of the dissolution tests were divided into two categories, soluble (+) and insoluble (−), based on visual inspection. If the samples were found to be completely dissolved, they were considered as soluble; if not, they were considered as insoluble.

### Swelling Ratio

Typically, a crosslinked polymer was weighed (around 30 mg) into a glass vial before adding THF (2 mL) at room temperature. After 24 h, the sample was retrieved, lightly blot dried, and weighed. The swelling ratio was calculated according to eq 1, in which *m*_1_ is the mass of the initial dry polymer and *m*_2_ is the mass of the material after swelling in THF.
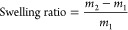
1

### Gel Content

A crosslinked polymer was weighed into a filter paper satchel and submerged in ethyl acetate (EtOAc). The solution was refluxed for 2 h, and the satchel was removed and washed with EtOAc. Next, the sample packet was dried in a vacuum oven at 100 °C for 2 h and weighed afterward. The gel content was calculated according to eq 2, where *m*_0_ is the mass of the satchel, *m*_1_ is the initial mass of the polymer sample, and *m*_3_ is the satchel weight after refluxing and drying.
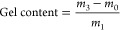
2

### Synthesis of Vinyl Levulinate

The synthesis was carried out according to a previously reported procedure.^62^ Levulinic acid (10.0 g, 86.1 mmol), vinyl acetate (79.4 mL, 861 mmol), KOH (483 mg, 8.61 mmol), and Pd(OAc)_2_ (479 mg, 2.13 mmol) were weighed into a 250 mL flask. The mixture was heated to 60 °C in an oil bath and stirred at this temperature for 23 h. Then the mixture was cooled to room temperature and filtered over Celite; petroleum ether was used for washing. The solvent was removed with a rotary evaporator, and the obtained crude product was further distilled under reduced pressure (70 °C, 5 mm Hg) using a Büchi Kugelrohr device with Glass Oven B-585 to obtain the product as a colorless oil (7.3 g, yield 60%). The NMR data of the obtained vinyl levulinate was consistent with the previous literature report.^62^

### Enzymatic Synthesis of 2-Levulinoyl-5-methacrylate-Isosorbide (IL)

**Figure 2:**
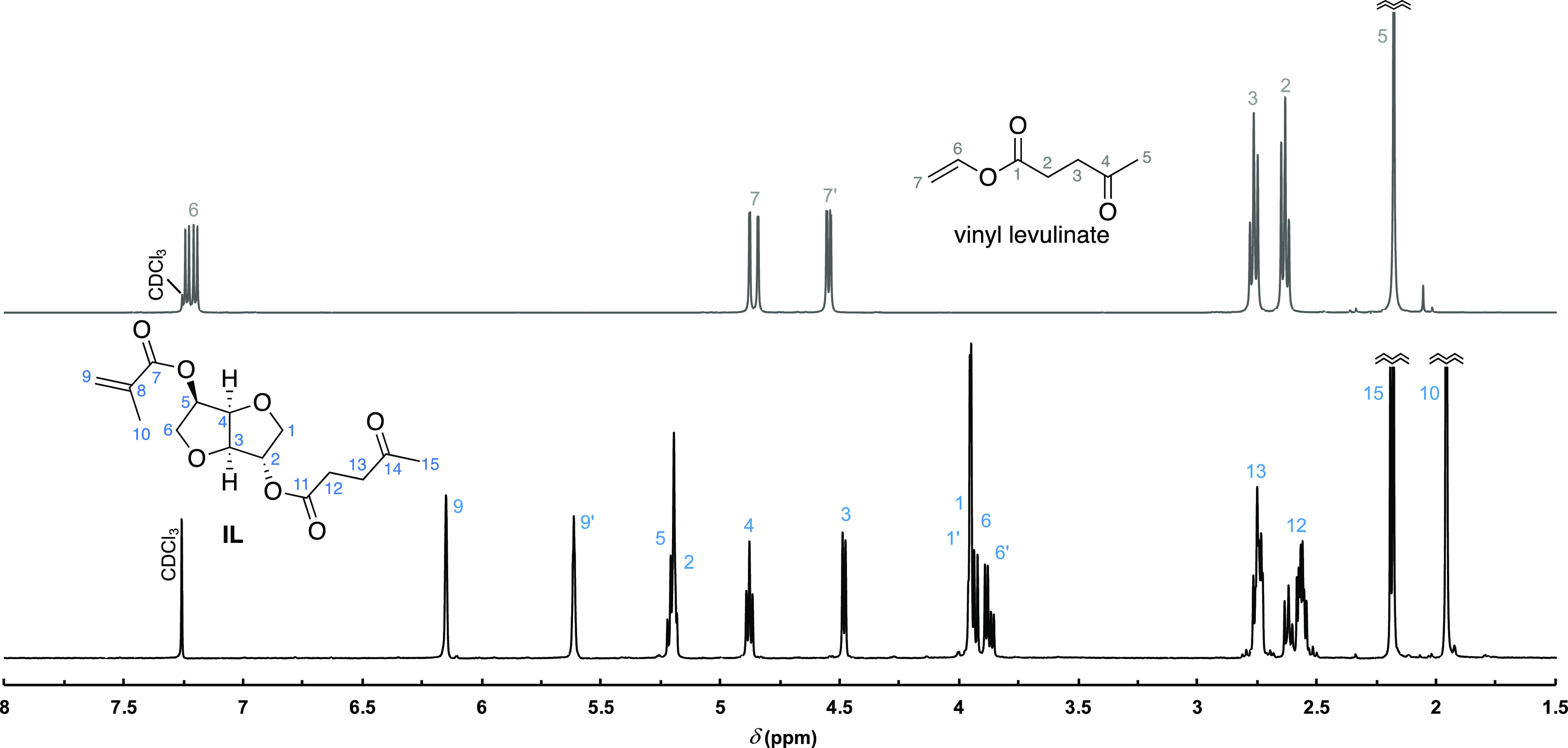
Crude ^1^H NMR spectrum of IL synthesized in 2-MeTHF after enzyme filtration and drying under reduced pressure (purity > 98%). ^1^H NMR spectrum of vinyl levulinate included for comparison.

5-Methacrylate-isosorbide (292 mg, 1.36 mmol), vinyl levulinate (0.29 mL, 2.05 mmol), 2-methyltetrahydrofuran (2-MeTHF, 4 mL), and Novozym 435 (*Candida antarctica* lipase B (CALB) immobilized on resin, 55 mg) were introduced into a 25 mL flask. The mixture was stirred slowly at room temperature. The experiment was monitored by TLC (5% CH_3_OH/CH_2_Cl_2_) and ^1^H NMR spectroscopy. After 40 h, the synthesis was terminated by filtering off the enzyme, the solvent was then removed by rotary evaporator, and the excess of vinyl levulinate was removed under reduced pressure (roughly 5 mbar) until no vinyl levulinate signals were detectable on the ^1^H NMR spectrum (typically around 48 h at room temperature, Figure 2). The product was obtained as a colorless oil (413 mg, yield 97%). In order to prevent polymerization during storage, the product was kept in an EtOAc solution with hydroquinone (HQ, 2 mg). The use of methyl *tert*-butyl ether (MTBE) and acetonitrile (ACN) as solvents was also evaluated under the same reaction conditions. **IL** was obtained in 96% yield in both cases (Figures S2 and S3).

TLC: *R*_f_ = 0.60 (5% CH_3_OH/CH_2_Cl_2_). IR (ATR) ν_max_ (cm^–1^): 1717, 1636, 1362, 1296, 1157, 1096. HRMS (nESI + FT-ICR-MS): calcd for C_15_H_20_O_7_Na [M + Na]^+^, 335.11013; found, 335.1101. ^1^H NMR (400.1 MHz, CDCl_3_): δ 6.15 (m, 1H), 5.61 (m, 1H), 5.20 (m, 2H), 4.88 (dm, *J* = 5.2 Hz, 1H), 4.48 (dm, *J* = 4.8 Hz, 1H), 3.94 (dm, *J* = 2.3 Hz, 2H), 3.90 (ddm, *J* = 10.0, 4.8 Hz, 2H), 2.74 (tdm, *J* = 6.5, 2.3 Hz, 2H), 2.56 (tdm, *J* = 6.5, 3.1 Hz, 2H), 2.18 (s, 3H), 1.95 (s, 3H) ppm. ^13^C NMR (100.6 MHz, CDCl_3_): δ 206.34, 171.86, 166.64, 135.68, 126.30, 85.93, 80.91, 78.18, 74.09, 73.19, 70.69, 37.81, 29.78, 27.92, 18.29 ppm.

### General Procedure for Free Radical Polymerization of Methacrylates

**IL** (70–470 mg, 0.05–1.0 equiv.) was filtered through basic Al_2_O_3_ to remove the inhibitor (HQ) and placed into an 8 mL pressure tube. In the cases where copolymers were prepared, **MMA** was also added (0.2–0.95 equiv., 0.04–0.47 mL). When poly(methylmethacrylate) (**PMMA**) was prepared, only **MMA** (1.0 equiv., 0.53 mL) was added into the pressure tube. **MMA** was also filtered through basic Al_2_O_3_ to remove the inhibitor (HQ) before use. Next, EtOAc (∼5 mL; the total monomer concentration was around *c* = 100 mg mL^–1^) and azobis(isobutyronitrile) (AIBN; 0.5 mol %) dissolved in EtOAc were added. After sparging the mixture with Ar for 30 min, the tube was sealed firmly with a cap and placed into a preheated oven at 63 °C for 24 h. The reaction was then cooled to room temperature, and a small sample was taken to determine the monomer conversion by ^1^H NMR spectroscopy. The crude product was precipitated in Et_2_O to remove any residual monomer and filtered off. Thereafter, the precipitate was washed twice more with an Et_2_O/isopropanol (5:1) mixture before collecting the solid product that was carefully dried in a vacuum oven at 40 °C. The linear copolymers were abbreviated as **P(MMA**_***x***_**-IL**_***y***_**)**, where ***x*** corresponds to the mol % of **MMA** and ***y*** corresponds to the mol % of **IL** in the specific copolymer. The dried product was characterized by ^1^H NMR, FTIR, SEC, TGA, and DSC measurements.

### Crosslinking of Linear Polymethacrylates with Dihydrazides

Linear polymethacrylate (50–100 mg) was transferred into a 5 mL round-bottom flask that was capped with a septum and purged with Ar for 10 min. The polymethacrylate was thereafter dissolved in γ-valerolactone (GVL) or 1,4-dioxane (0.5–1.0 mL), before adding a droplet of acetic acid (0.02 mL) into the flask. Next, a specified amount (2.5–40 mol %) of **adh** or **mdh** was weighed into a small vial. The amount of the dihydrazide used corresponded to half of the molar **IL** content in the linear copolymer, as one dihydrazide molecule reacts with two ketone groups. Next, the dihydrazide was dissolved in H_2_O (0.1–0.2 mL) and added dropwise via a syringe to the linear polymethacrylate in GVL or 1,4-dioxane. The reaction was stopped when the mixture turned into a very viscous gel and the magnetic stirrer bar was no longer moving (usually after around 5 min). If the mixture did not turn gelly, it was let to stir for a longer time (up to 24 h). The product was then precipitated in an Et_2_O/isopropanol (5:1) mixture and filtered off. Thereafter, the precipitate was washed twice more with the Et_2_O/isopropanol mixture. Then a solid product was collected and dried in a vacuum oven at 60 °C. The crosslinked copolymers were abbreviated as **P(MMA**_***x***_**-IL**_***y***_**)-adh**_***z***_ or **P(MMA**_***x***_**-IL**_***y***_**)-mdh**_***z***_, where ***z*** corresponds to the mol % of corresponding crosslinker used. The dried product was characterized by FTIR, TGA, and DSC measurements.

### De-crosslinking of Dihydrazide-crosslinked Polymers

In a typical experiment, acetone-*d*_6_ (0.9 mL) and D_2_O solutions (0.1 mL, pH = 1 or 4) were introduced into an NMR tube. The ^1^H NMR spectrum of the solution was recorded. The pH = 1 solution was prepared from D_2_O (2 mL) and 35% DCl solution in D_2_O (20 μL), and the pH = 4 solution was prepared from D_2_O (5 mL), sodium acetate (9.3 mg), and acetic acid (22 μL). Next, a dihydrazide-crosslinked polymer sample (20 mg) was added into the same NMR tube and the ^1^H NMR spectra were measured after certain intervals. In the case of acidic D_2_O of pH = 1, the spectra were measured more frequently as the de-crosslinking in this environment took less time than in the solution with acidic D_2_O of pH = 4. The decomposition of the crosslinked network could be seen visually by the dissolution of the solid polymer in the NMR tube and in the spectra by increasing the signal intensities. At first, the crosslinked polymer was completely insoluble in the acetone-*d*_6_–D_2_O medium. However, as the crosslinks were cleaved in the polymer structure, the polymer became increasingly soluble and visible in the ^1^H NMR spectra. The experiment was stopped when the integrals in ^1^H NMR spectra had not changed for at least 10 min. The mixture in the NMR tube was then poured into an Et_2_O/isopropanol (5:1) mixture to precipitate the sample. After filtering off the precipitate, it was washed twice more with the Et_2_O/isopropanol mixture, before it was collected and dried in a vacuum oven at 40 °C. The dried product [***de***_***q***_**-P(MMA**_***x***_**-IL**_***y***_**)-adh**_***z***_] was characterized by ^1^H NMR, FTIR, SEC, TGA, and DSC measurements. The ^1^H NMR and FTIR data of the de-crosslinked polymers (Figures S12–S17, S22) were consistent with that of the corresponding linear polymethacrylates.

### Re-crosslinking of the De-crosslinked Copolymer

This experiment was carried out in a similar way as the crosslinking of the primary linear polymethacrylates with dihydrazides described above. A de-crosslinked linear polymethacrylate ***de***_***1***_***-*P(MMA**_**80**_**-IL**_**20**_**)-adh**_**10**_ (20 mg) was transferred into a 5 mL round-bottom flask that was capped with a septum and purged with Ar for 10 min. The polymethacrylate was dissolved in GVL (0.15 mL), and a droplet of acetic acid (0.01 mL) was added into the flask. Next, **adh** (2.3 mg, 10 mol %; this corresponded to half the molar content of 2-levulinoyl-5-methacrylate-isosorbide in this polymer) was weighed into a small vial and dissolved in H_2_O (0.03 mL). Then the dihydrazide in H_2_O was added dropwise via a syringe to the polymethacrylate in GVL. The reaction was stopped after 24 h, and the product was then precipitated into an Et_2_O/isopropanol (5:1) mixture and filtered off. Thereafter, the obtained precipitate was washed twice more with the Et_2_O/isopropanol mixture. Then the solid product was collected and dried in a vacuum oven at 60 °C. The dried product ***re*-P(MMA**_**80**_**-IL**_**20**_**)-adh**_**10**_ was characterized by FTIR, TGA, and DSC measurements.

## Results and Discussion

### Synthesis of Levulinic Isosorbide Monomethacrylate

**Scheme 1:**
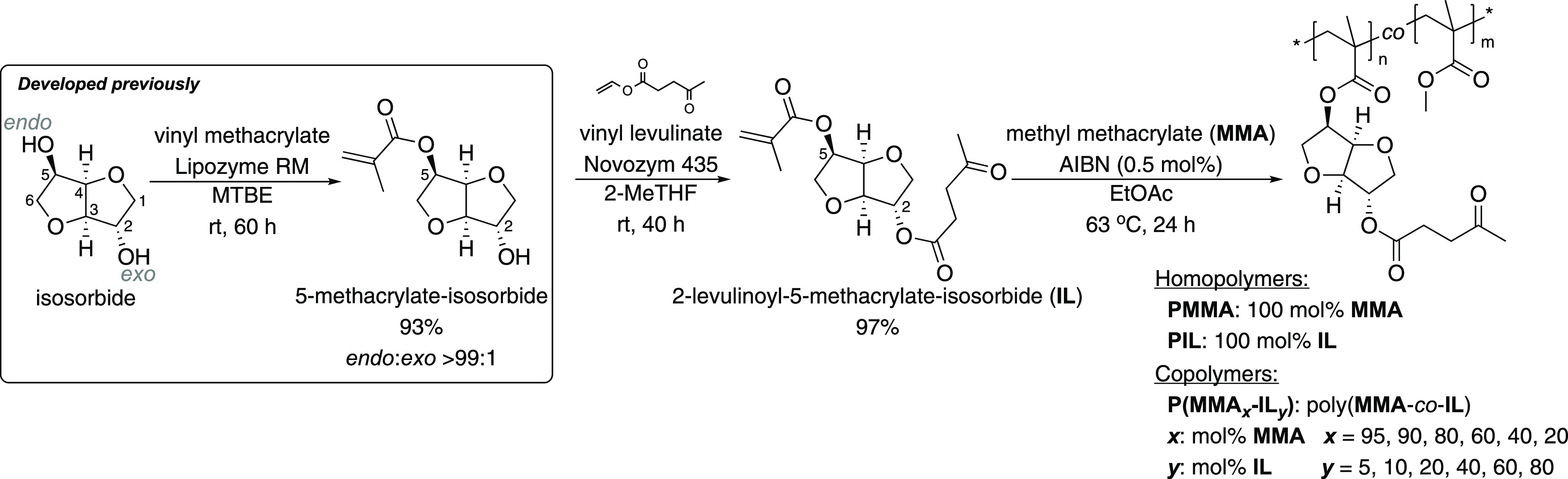
Biocatalytic Synthesis of 5-Methacrylate Isosorbide With Lipozyme RM and Vinyl Methacrylate,^16^ Followed by the Biocatalytic Synthesis of Levulinic Isosorbide Monomethacrylate (IL) With Novozym 435 and Vinyl Levulinate, and the Radical Copolymerization of IL and MMA

The levulinic isosorbide monomethacrylate monomer was synthesized in a two-step chromatography-free procedure in 90% yield from commercial-grade isosorbide (Scheme 1). First, the isosorbide was monomethacrylated with vinyl methacrylate using immobilized *Rhizomucor miehei* (RM) lipase as a biocatalyst (lipozyme RM), in >99% regioselectivity and 93% yield, as reported previously by our group.^16^ In the second step, 5-methacrylate was reacted with vinyl levulinate in the presence of immobilized *C. antarctica* lipase B (Novozym 435) as the acyl transfer catalyst. Vinyl levulinate was conveniently prepared from levulinic acid and vinyl acetate in a chromatography-free process using the Pd(OAc)_2_/KOH catalytic system as reported before.^62^ We have previously demonstrated that Novozym 435 can be used to catalyze the esterification of 2-OH in isosorbide.^15,16^

In our first attempt, the reaction of 5-methacrylate-isosorbide with vinyl levulinate was carried out in ACN, and the desired 2-levulinoyl-5-methacrylate-isosorbide (**IL**) was obtained in 85% yield after purification by short column chromatography. Next, we switched to a greener solvent (2-MeTHF) and tested the chromatography-free approach. The reaction mixture was gently stirred in 2-MeTHF at room temperature, and the reaction progress was monitored by TLC. After 40 h, no 5-methacrylate-isosorbide could be detected. The enzyme was filtered off, and the crude product was dried under reduced pressure to remove the solvent and the excess of vinyl levulinate to afford the **IL** in 97% yield. The ^1^H NMR analysis indicated only traces of unknown impurities (Figure 2). Besides the biocatalytic synthesis of **IL**, we also carried out conventional chemical acylation of 5-methacrylate-isosorbide. This experiment was conducted with levulinoyl chloride^63^ and Et_3_N in CH_2_Cl_2_. After stirring the mixture at ambient temperature for 18 h, followed by extractive workup and purification by column chromatography, the target **IL** was obtained in 66% yield, i.e., lower in comparison to the enzymatic method.

The ketone functionality present in the monomer side chain offers a handle for additional transformations like ketalization reactions, reduction to alcohols, reactions with hydrazides to obtain acylhydrazones, etc. Consequently, we have developed a multifunctional **IL** monomer from commercial isosorbide via a high-yielding, chromatography-free, and easily upscalable biocatalytic procedure.

### Radical (Co)Polymerization of Levulinoyl-isosorbide-monomethacrylate with Methyl Methacrylate and Characterization of the Products

Next, we turned our attention to the preparation of (co)polymers of **IL** and **MMA** via conventional radical polymerization with AIBN in ethyl acetate at 63 °C, as seen in Scheme 1. The combined monomer concentration was 100 mg mL^–1^, and the initiator concentration was 0.5 mol %. After 24 h, ^1^H NMR spectra of the reaction mixtures were recorded to determine the conversion of monomers. Next, the crude polymers were purified by precipitation in a 5:1 (v/v) mixture of Et_2_O and isopropanol, before filtering off the solid precipitates. The obtained white polymer powders were further dried under reduced pressure at 40 °C to remove solvent residues, before confirming the polymer structures by ^1^H NMR spectroscopy. The resulting statistical copolymers are named as **P(MMA**_***x***_**-IL**_***y***_**)**, where ***x*** corresponds to the mol % of **MMA** and ***y*** corresponds to the mol % of **IL** in the specific copolymer. The ratio of **IL** in the copolymers was varied from 5 to 80 mol % (Table 1, entries 2–7). Additionally, the **PMMA** and **PIL** homopolymers were prepared from **MMA** and **IL**, respectively (Table 1, entries 1 and 8), as reference materials.

**Table 1:** Polymerization and Thermal Data of the Linear Polymethacrylates

		monomer target ratio (mol %)		monomer conversion (mol %)^a^		monomer ratio in dried polymer (mol %)^b^					
entry	polymethacrylate	MMA	IL	MMA	IL	MMA	IL	*M*_n_ (kg mol^–^^1^)^c^	D̵^c^	*T*_d,95_ (°C)^d^	*T*_g_ (°C)^e^
1	**PMMA**	100	0	72		100	0	31.6	1.5	246	127
2	**P(MMA**_**95**_**-IL**_**5**_**)**	95	5	68	78	95	5	32.1	1.6	206	115
3	**P(MMA**_**90**_**-IL**_**10**_**)**	90	10	73	78	90	10	49.1	1.6	206	95
4	**P(MMA**_**80**_**-IL**_**20**_**)**	80	20	57	72	81	19	36.8	1.6	222	96
5	**P(MMA**_**60**_**-IL**_**40**_**)**	60	40	63	82	57	43	26.8	1.6	203	89
6	**P(MMA**_**40**_**-IL**_**60**_**)**	40	60	59	82	37	63	20.4	1.7	217	75
7	**P(MMA**_**20**_**-IL**_**80**_**)**	20	80	58	75	21	79	16.6	1.6	209	77
8	**PIL**	0	100		81	0	100	42.1	2.0	210	74

a Determined by ^1^H NMR spectroscopy of the crude polymer.

b Determined by ^1^H NMR spectroscopy of the dried polymer.

c Determined by SEC in THF using poly(methyl methacrylate) standards.

d Determined by TGA at 5% weight loss under N_2_.

e Determined by DSC.

As can be seen in Table 1, the conversions of the monomers in the (co)polymerizations, evaluated from crude ^1^H NMR spectra, varied between 72 and 82% in the case of **IL**, and in the range 57–73% for **MMA**. The monomer ratios of the dried copolymers corresponded quite accurately to the monomer feed ratios according to the ^1^H NMR spectra of the isolated copolymers. In the case of **P(MMA**_**80**_**-IL**_**20**_**)** and **P(MMA**_**20**_**-IL**_**80**_**)**, the difference was 1 mol % in comparison to the monomer feed ratio, and with **P(MMA**_**60**_**-IL**_**40**_**)** and **P(MMA**_**40**_**-IL**_**60**_**)**, the variation was 3 mol %. The number average molecular weights (*M*_n_) and dispersity (D̵) were determined by SEC in THF, as presented in Figure S24. The *M*_n_ values of these linear copolymers varied from 16.6 kg mol^–1^ [**P(MMA**_**20**_**-IL**_**80**_**)**, Table 1, entry 7] to 49.1 kg mol^–1^ [**P(MMA**_**90**_**-IL**_**10**_**)**, Table 1, entry 3], and *D̵* ranged from 1.5 to 2.0. These results are comparable to the methacrylic isosorbide polymers previously prepared by our group in a similar way.^15,16^

The solubility of the linear copolymers was determined in a range of solvents selected based on their solubility parameter (δ) and hydrogen-bonding capacity (Table S1). All the polymethacrylates were insoluble in strongly hydrogen-bonding solvents like H_2_O, CH_3_OH, and *n*-butanol (*n*-BuOH). Among the solvents with moderate hydrogen-bonding capacity, DMSO (dimethyl sulfoxide, δ = 25 MPa^1/2^) and THF (δ = 19 MPa^1/2^) dissolved the polymethacrylates, while Et_2_O (δ = 15 MPa^1/2^) did not. In addition, ACN (δ = 24 MPa^1/2^) and CHCl_3_ (δ = 19 MPa^1/2^) with similar solubility parameter values as DMSO and THF, respectively, also dissolved these copolymers. In the case of toluene (δ = 18 MPa^1/2^), the solubility depended on the levulinic monomer content. Copolymers with lower **IL** content were soluble in toluene, while copolymers with an **IL** content from 40 to 100 mol % [(**P(MMA**_**60**_**-IL**_**40**_**)**, **P(MMA**_**40**_**-IL**_**60**_**)**, and **P(MMA**_**20**_**-IL**_**80**_**)**, **PIL**] were insoluble in this medium.

**Figure 3:**
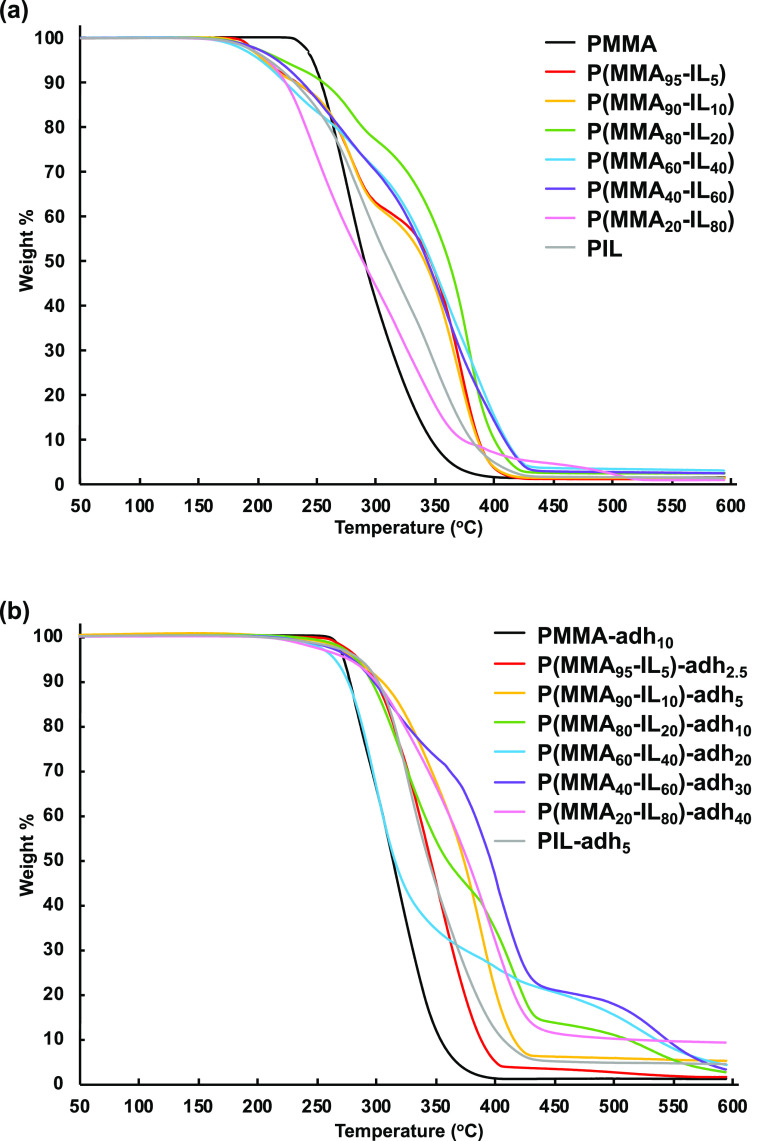
TGA curves of the linear polymethacrylates (a) and the corresponding **adh**-crosslinked samples (b).

The thermal stability of the polymers was analyzed by TGA under nitrogen at a heating rate of 10 °C min^–1^ (Figure 3a). All the linear polymethacrylates exhibited thermal decomposition temperature (*T*_d,95_) above 200 °C, determined at 5% weight loss. The highest *T*_d,95_ was determined for **PMMA** (246 °C). For the isosorbide-based homopolymer **PIL**, the *T*_d,95_ was lower (210 °C). For the copolymers, the *T*_d,95_ varied between 203 and 222 °C. Generally, increasing the **IL** content in the copolymer decreases the *T*_d,95_ (Table 1). Plots of the decomposition rate (DTG profiles, Figure S27b) indicated a multistep degradation for all the linear (co)polymers, except for **PMMA**. Hence, this multistep process was most probably caused by the presence of the levulinic isosorbide side chains in these polymers. We speculate that the first weight loss was caused by the loss of the flexible levulinic segment, followed by the decomposition of the isosorbide unit, and finally, the slow degradation of the polymer main chain.

**Figure 4:**
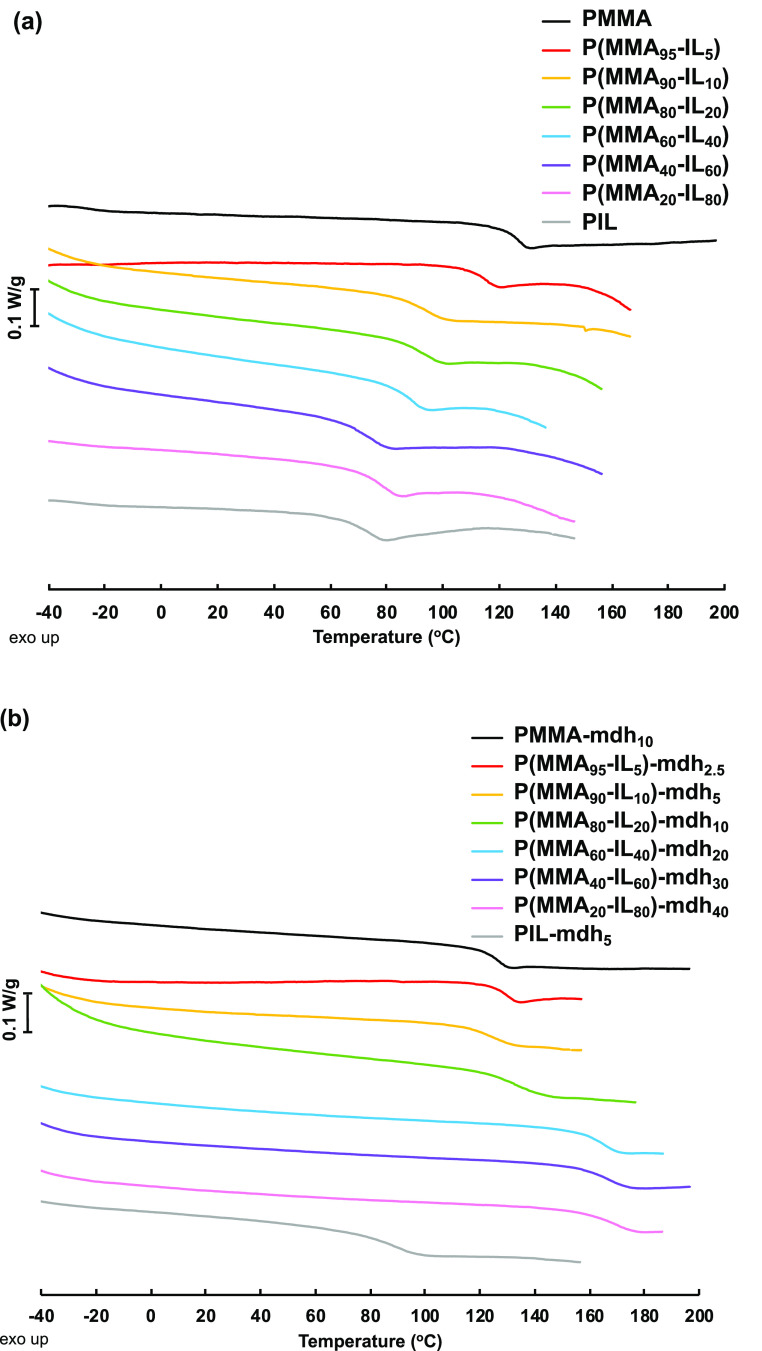
DSC second heating curves of the linear polymethacrylates (a) and the corresponding **mdh**-crosslinked samples (b).

The thermal behavior of polymethacrylates was analyzed by DSC to study the glass transitions (Figure 4a). As seen in Table 1, the *T*_g_ of **PIL** was 74 °C (entry 8), and it gradually increased with increasing **MMA** content of the copolymers. Hence, the highest *T*_g_ was determined for **PMMA** at 127 °C (Table 1, entry 1). This value was higher than that typically reported for **PMMA** (105 °C).^64^ This may be explained by the slightly syndiotactic-rich configuration of the present **PMMA** (*rr* = 61%, Figure S4), which was a result of the free-radical polymerization used in our study. This kind of *T*_g_ increase due to the syndiotactic-rich configuration is well-known.^65^ In **MMA** copolymers, the preference toward syndiotactic-rich configuration was maintained (Figures S6–S11).

### Crosslinking of Linear Polymethacrylates with Dihydrazides

**Scheme 2:**
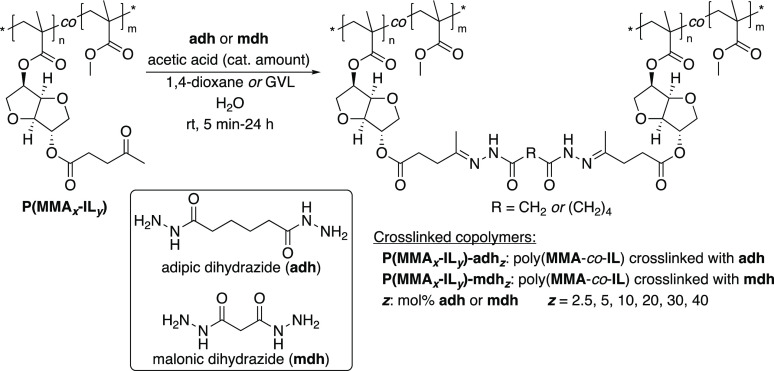
Post-polymerization Crosslinking of Levulinic-Isosorbide Polymethacrylates With Dihydrazides

With the linear polymethacrylates at hand, we next studied the crosslinking reaction between dihydrazides and the ketone group present in the levulinic side chain of these polymers (Scheme 2 and Table 2). Two dihydrazides with different chain lengths were evaluated, i.e., **adh** (6 carbon chain) and **mdh** (3 carbon chain) (Scheme 2). The crosslinked copolymers are designated as **P(MMA**_***x***_**-IL**_***y***_**)-adh**_***z***_ or **P(MMA**_***x***_**-IL**_***y***_**)-mdh**_***z***_, where the ***z*** denotes the concentration (mol %) of **adh** or **mdh**, respectively (Table 2, entries 2–10 and 13–18). The amount of dihydrazide used was related to the ketone content in the linear copolymer, taking into account that one dihydrazide reacts with two ketone groups (e.g., if the polymer contained 40 mol % levulinic monomer, then 20 mol % of dihydrazide was used). In the case of the homopolymer of **PIL**, 5 mol % of **adh** or **mdh** was added (Table 2, entries 11 and 19), and in the crosslinking attempts of **PMMA** (control experiments), 10 mol % of either of the dihydrazides was used (Table 2, entries 1 and 12).

**Table 2:** Synthesis and Thermal Data of the Dihydrazide-crosslinked Polymethacrylates

entry	linear polymethacrylate	crosslinking unit with amount (mol %)	solvent^a^	reaction time	crosslinked polymer^b^	*T*_d,95_ (°C)^c^	*T*_g_ (°C)^d^	swelling ratio^e^
1	**PMMA**	**adh**_**10**_	GVL/H_2_O	24 h	**PMMA-adh**_**10**_	273	126	n.d*.*^f^
2	**P(MMA**_**95**_**-IL**_**5**_**)**	**adh**_**2.5**_	GVL/H_2_O	19 h	**P(MMA**_**95**_**-IL**_**5**_**)-adh**_**2.5**_	286	125	n.d*.*^f^
3	**P(MMA**_**90**_**-IL**_**10**_**)**	**adh**_**5**_	1,4-dioxane/H_2_O	5 min	**P(MMA**_**90**_**-IL**_**10**_**)-adh**_**5**_	282	109	3.2
4	**P(MMA**_**80**_**-IL**_**20**_**)**	**adh**_**2.5**_	GVL/H_2_O	24 h	**P(MMA**_**80**_**-IL**_**20**_**)-adh**_**2.5**_	275	102	n.d*.*^f^
5	**P(MMA**_**80**_**-IL**_**20**_**)**	**adh**_**5**_	GVL/H_2_O	24 h	**P(MMA**_**80**_**-IL**_**20**_**)-adh**_**5**_	277	107	n.d*.*^f^
6	**P(MMA**_**80**_**-IL**_**20**_**)**	**adh**_**10**_	1,4-dioxane/H_2_O	5 min	**P(MMA**_**80**_**-IL**_**20**_**)-adh**_10_*	285	129	n.d*.*^f^
7	**P(MMA**_**80**_**-IL**_**20**_**)**	**adh**_**10**_	GVL/H_2_O	5 min	**P(MMA**_**80**_**-IL**_**20**_**)-adh**_**10**_	283	117	1.9
8	**P(MMA**_**60**_**-IL**_**40**_**)**	**adh**_**20**_	1,4-dioxane/H_2_O	5 min	**P(MMA**_**60**_**-IL**_**40**_**)-adh**_**20**_	265	125	2.9
9	**P(MMA**_**40**_**-IL**_**60**_**)**	**adh**_**30**_	1,4-dioxane/H_2_O	5 min	**P(MMA**_**40**_**-IL**_**60**_**)-adh**_**30**_	279	138	3.2
10	**P(MMA**_**20**_**-IL**_**80**_**)**	**adh**_**40**_	1,4-dioxane/H_2_O	5 min	**P(MMA**_**20**_**-IL**_**80**_**)-adh**_**40**_	276	129	2.3
11	**PIL**	**adh**_**5**_	GVL/H_2_O	30 min	**PIL-adh**_**5**_	273	72	6.6
12	**PMMA**	**mdh**_**10**_	GVL/H_2_O	24 h	**PMMA-mdh**_**10**_	281	127	n.d*.*^f^
13	**P(MMA**_**95**_**-IL**_**5**_**)**	**mdh**_**2.5**_	GVL/H_2_O	10 h	**P(MMA**_**95**_**-IL**_**5**_**)-mdh**_**2.5**_	264	130	4.5
14	**P(MMA**_**90**_**-IL**_**10**_**)**	**mdh**_**5**_	GVL/H_2_O	5 min	**P(MMA**_**90**_**-IL**_**10**_**)-mdh**_**5**_	230	123	3.6
15	**P(MMA**_**80**_**-IL**_**20**_**)**	**mdh**_**10**_	GVL/H_2_O	5 min	**P(MMA**_**80**_**-IL**_**20**_**)-mdh**_**10**_	235	133	2.3
16	**P(MMA**_**60**_**-IL**_**40**_**)**	**mdh**_**20**_	GVL/H_2_O	5 min	**P(MMA**_**60**_**-IL**_**40**_**)-mdh**_**20**_	223	165	3.9
17	**P(MMA**_**40**_**-IL**_**60**_**)**	**mdh**_**30**_	GVL/H_2_O	5 min	**P(MMA**_**40**_**-IL**_**60**_**)-mdh**_**30**_	229	166	2.7
18	**P(MMA**_**20**_**-IL**_**80**_**)**	**mdh**_**40**_	GVL/H_2_O	5 min	**P(MMA**_**20**_**-IL**_**80**_**)-mdh**_**40**_	236	170	3.0
19	**PIL**	**mdh**_**5**_	GVL/H_2_O	30 min	**PIL-mdh**_**5**_	251	92	3.2

a Solvent mixture with a 4:1 (v/v) ratio in all cases.

b The name of the crosslinked polymer is presented as **P(MMA**_***x***_**-IL**_***y***_**)-adh**_***z***_ or **P(MMA**_***x***_**-IL**_***y***_**)-mdh**_***z***_, in which ***z*** corresponds to the concentration of **adh** or **mdh** in mol %, respectively.

c Determined by TGA at 5% weight loss under N_2_.

d Determined by DSC.

e Determined in THF.

f n.d.—not determined.

The post-polymerization modifications were first evaluated in a 4:1 (v/v) mixture of 1,4-dioxane and H_2_O. This solvent mixture was chosen because it dissolves both the linear polymers and the dihydrazides, and it has also previously been successfully used for acylhydrazone bond formation.^66,67^ Alternative solvents (e.g., H_2_O,^43^ ethanol,^68,69^ methanol,^44^ and isopropanol^70^) reported for acylhydrazone bond formation did not dissolve the present isosorbide-based linear polymethacrylates (Table S1). 1,4-Dioxane is known for its unfavorable environmental profile and is harmful to human health.^71^ Consequently, we replaced 1,4-dioxane with GVL, which is considered a greener alternative.^72^ GVL readily dissolved the linear polymethacrylates just as efficiently as 1,4-dioxane. Moreover, the influence of the solvent change was evaluated by carrying out two crosslinking experiments between **P(MMA**_**80**_**-IL**_**20**_**)** and **adh** in these two different solvent mixtures (Table 2, entries 6 and 7). Since similar thermal data were determined for these two crosslinked networks, it was concluded that the solvent change did not have any significant influence on the final **adh**-crosslinked polymethacrylates. Thus, the solvent switch to a greener alternative was successful.

The reaction mixtures in the crosslinking experiments were stirred at ambient temperature until they changed to a very thick gel, which stopped the magnetic stirrer bar. In general, it took around 5 min, but in some cases, the change was observed after 30 min. If the gel formation was not visually observed within 30 min, the reaction mixture was left to stir for longer (up to 24 h). Thereafter, the crude crosslinked network was precipitated into a 5:1 (v/v) Et_2_O/isopropanol mixture. The precipitate was then filtrated and dried under reduced pressure at 60 °C to obtain the product as a white solid powder.

As expected, all corresponding dihydrazide-crosslinked (co)polymers were insoluble in all the tested solvents (Table S1), which was in contrast to the corresponding linear polymethacrylates. FTIR analysis of the dried crosslinked samples indicated the presence of a C=N bond (see Figures 6b, S20, and S21). Additionally, swelling ratios of the crosslinked polymers in THF were found to vary between 1.9 and 6.6. The insolubility and the swelling behavior further proved the formation of crosslinked networks. But we did not find any clear trend between the swelling ratio and the concentration of the dihydrazide used. Also, the swelling ratio did not seem to depend on the hydrazide length (**adh** or **mdh**). We speculate that the reason behind these peculiarities may be the differences in *M*_n_ values of linear prepolymers used in crosslinking experiments. The gel content was also investigated and was found to be over 90% for all the crosslinked polymers; this value was not dependent on crosslinker amount.

TGA measurements (Figures 3b, S28, and S30a) of the **adh**- and **mdh**-crosslinked polymers indicated an approximate 10–80 °C increase of *T*_d,95_ in comparison to the corresponding linear polymethacrylates. The largest change occurred in the case of **P(MMA**_**95**_**-IL**_**5**_**)-adh**_**2.5**_ whose *T*_d,95_ value (286 °C) was 80° higher than that of the linear **P(MMA**_**95**_**-IL**_**5**_**)** (*T*_d,95_ = 206 °C). The smallest increase of 7 °C was observed for **P(MMA**_**80**_**-IL**_**20**_**)-mdh**_**10**_ (*T*_d,95_ = 235 °C) in comparison with the linear **P(MMA**_**80**_**-IL**_**20**_**)** (*T*_d,95_ = 222 °C). Thus, the effect of crosslinking can be clearly seen from these *T*_d,95_ values. Similar to the linear polymers, the crosslinked polymers also showed a multistep degradation in their DTG profiles (Figures S29 and S30b).

The thermal behavior of the crosslinked polymers, as characterized by DSC analysis, also demonstrated an increase in *T*_g_s in comparison to the corresponding linear polymethacrylates (Figures 4b and S34). As expected, the *T*_g_ increased with the dihydrazide concentration in the copolymer network. Still, the *T*_g_ of **P(MMA**_**40**_**-IL**_**60**_**)-adh**_**30**_ was slightly higher than the *T*_g_ of the following **P(MMA**_**20**_**-IL**_**80**_**)-adh**_**40**_, which can be explained by the higher *M*_n_ of the linear polymethacrylate **P(MMA**_**40**_**-IL**_**60**_**)** used for the synthesis of this crosslinked network. Furthermore, the impact of the carbon chain length in the dihydrazide structure was also evident, as the use of the shorter **mdh** with a C3 chain increased the *T*_g_ much more compared to polymers crosslinked with the longer and more flexible **adh**. For instance, the *T*_g_s values of **P(MMA**_**60**_**-IL**_**40**_**)-mdh**_**20**_ (165 °C) and **P(MMA**_**20**_**-IL**_**80**_**)-mdh**_**40**_ (170 °C), with **mdh** as the crosslinking agent, were about 40 °C higher than those for the corresponding **adh**-crosslinked polymers [*T*_g_ values of **P(MMA**_**60**_**-IL**_**40**_**)-adh**_**20**_ and **P(MMA**_**20**_**-IL**_**80**_**)-adh**_**40**_ were 125 and 129 °C, respectively].

The concentration of the crosslinker in the copolymers also influenced the thermal behavior. Three **adh**-crosslinked polymers [Table 2, entries 4, 5, and 7, polymers **P(MMA**_**80**_**-IL**_**20**_**)-adh**_**2.5**_, **P(MMA**_**80**_**-IL**_**20**_**)-adh**_**5**_, and **P(MMA**_**80**_**-IL**_**20**_**)-adh**_**10**_, respectively] were prepared from the same prepolymer **P(MMA**_**80**_**-IL**_**20**_**)** but with different crosslinker concentrations. Here, a slight increase of the *T*_g_ (102, 107, 117 °C, respectively) value was observed with increasing **adh** content in these crosslinked networks.

In control experiments, the **PMMA** homopolymer was subjected to the same crosslinking conditions (Table 2, entries 1 and 12). As expected, the attempted crosslinking of **PMMA** with 10 mol % of **adh** and 10 mol % of **mdh** did not result in crosslinking, as can be seen from the thermal data. The *T*_g_ of 126 °C for **PMMA-adh**_**10**_ and the *T*_g_ of 127 °C for **PMMA-mdh**_**10**_ are comparable to the corresponding value of the linear **PMMA** (*T*_g_ of 127 °C, Table 1, entry 1). Moreover, the solubility of **PMMA-adh**_**10**_ and **PMMA-mdh**_**10**_ were identical to the linear homopolymer **PMMA** (Table S1). Hence, no crosslinking occurred in the case of **PMMA**.

### De-crosslinking of Dihydrazide-crosslinked Polymethacrylates

**Figure 5:**
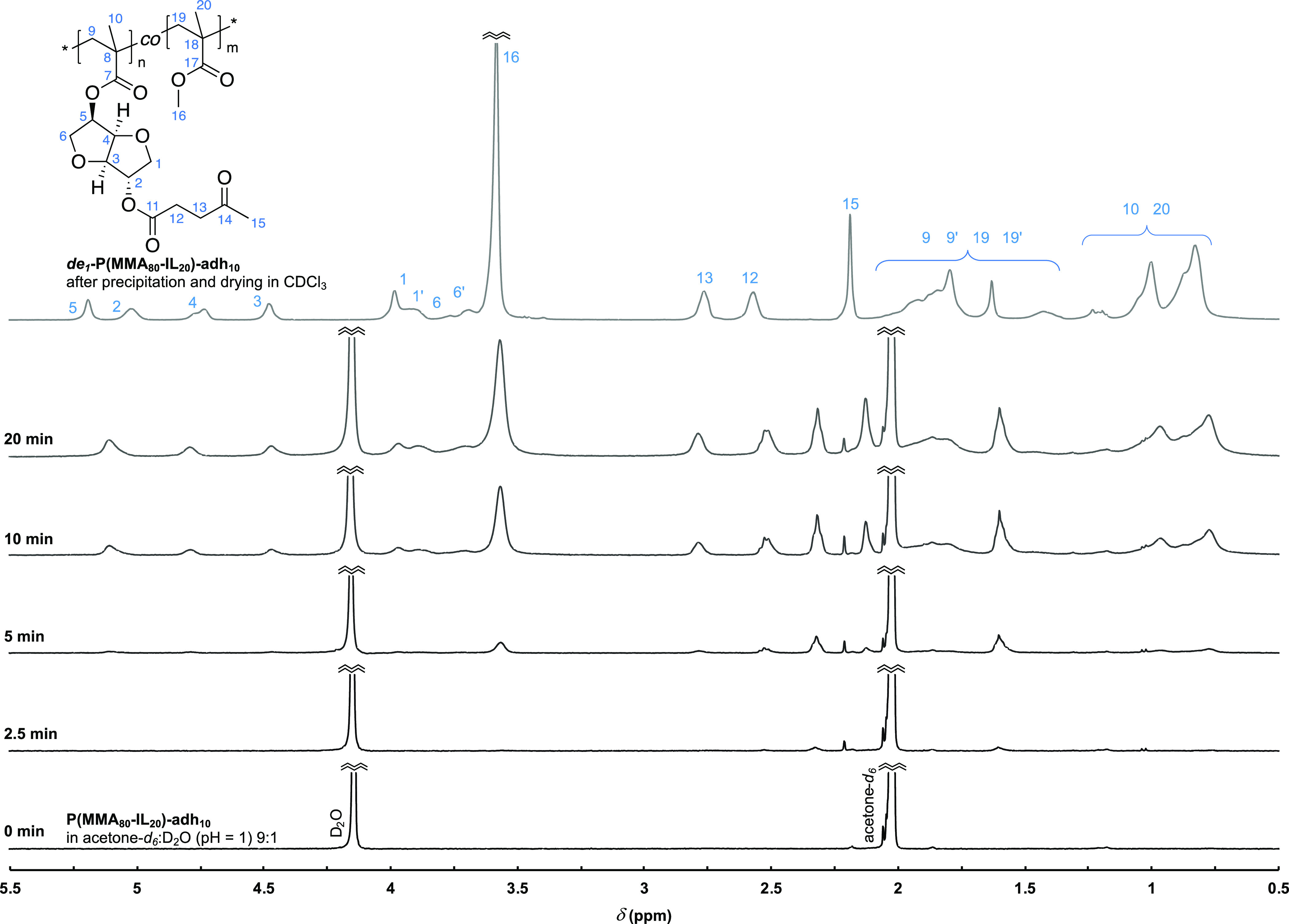
Sequence of ^1^H NMR spectra recorded up to 20 min after the start of the de-crosslinking of the **adh**-crosslinked polymer network in a 9:1 (v/v) acetone-*d*_6_/D_2_O mixture at pH = 1.

**Scheme 3:**
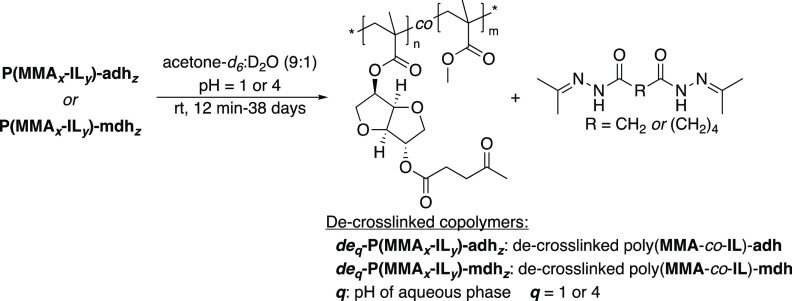
De-crosslinking of Dihydrazide-crosslinked Polymer Networks in Acetone-*d*_6_/D_2_O (9:1, v/v) At pH = 1 or 4

The acylhydrazone bond in the crosslinked polymer structure is a dynamic covalent bond. Consequently, we studied the removal of the crosslinking unit under acidic conditions (Scheme 3 and Table 3). The de-crosslinking is facilitated by the addition of a scavenging agent, which consumes the free amine and promotes the completion of the de-crosslinking reaction.^73^ Acetone is a widely used scavenging agent,^43^ and the use of acetone-*d*_6_ makes it possible to monitor the reaction in real time by NMR spectroscopy. Thus, the crosslinked polymer networks [**P(MMA**_***x***_**-IL**_***y***_**)-adh**_***z***_ or **P(MMA**_***x***_**-IL**_***y***_**)-mdh**_***z***_] were placed into a 9:1 (v/v) mixture of acetone-*d*_6_ and D_2_O, where the latter was acidified to pH of 1 or 4. These acidic solutions were prepared from D_2_O and a 35% DCl solution in D_2_O in the case pH = 1, and from a mixture of sodium acetate, acetic acid, and D_2_O to reach pH = 4. Initially, the solid polymer powder was insoluble when immersed into the mixture of acetone-*d*_6_ and acidic D_2_O. However, after some time, the polymer swelled and gradually started to dissolve until a transparent solution was formed. This change was observed by ^1^H NMR spectroscopy after specific time intervals (Figure 5). The de-crosslinking was considered complete when all the solid particles were dissolved, and no changes could be seen in the ^1^H NMR spectra. Notably, de-crosslinking in the more acidic medium (pH = 1) took considerably less time (no longer than 20 min) in comparison to the experiments carried out in the less acidic environment at pH = 4 (from 12 to 38 days). The de-crosslinked polymethacrylate samples are abbreviated as ***de***_***q***_**-P(MMA**_***x***_**-IL**_***y***_**)-adh**_***z***_ or ***de***_***q***_**-P(MMA**_***x***_**-IL**_***y***_**)-mdh**_***z***_, where ***q*** denotes the pH of the acidic solution.

**Table 3:** Reaction Conditions, Molecular Weights, and Thermal Data of the Linear De-crosslinked Polymethacrylates

entry	crosslinked copolymer	pH of acidic D_2_O solution	time	de-crosslinked polymethacrylate	*M*_n_ (kg mol^–^^1^)^a^	D̵^a^	*T*_d,95_ (°C)^b^	*T*_g_ (°C)^c^
1	**P(MMA**_**80**_**-IL**_**20**_**)-adh**_**10**_	1	20 min	***de***_***1***_**-P(MMA**_**80**_**-IL**_**20**_**)-adh**_**10**_	46.7	1.3	246	90
2	**P(MMA**_**80**_**-IL**_**20**_**)-adh**_**10**_	4	12 days	***de***_***4***_**-P(MMA**_**80**_**-IL**_**20**_**)-adh**_**10**_	40.9	1.5	267	90
3	**P(MMA**_**40**_**-IL**_**60**_**)-adh**_**30**_	1	20 min	***de***_***1***_**-P(MMA**_**40**_**-IL**_**60**_**)-adh**_**30**_	22.0	1.6	246	92
4	**P(MMA**_**40**_**-IL**_**60**_**)-adh**_**30**_	4	17 days	***de***_***4***_**-P(MMA**_**40**_**-IL**_**60**_**)-adh**_**30**_	20.7	1.8	248	57
5	**P(MMA**_**80**_**-IL**_**20**_**)-mdh**_**10**_	1	12 min	***de***_***1***_**-P(MMA**_**80**_**-IL**_**20**_**)-mdh**_**10**_	47.6	1.5	242	109
6	**P(MMA**_**40**_**-IL**_**60**_**)-mdh**_**30**_	4	38 days	***de***_***4***_**-P(MMA**_**40**_**-IL**_**60**_**)-mdh**_**30**_	16.4	2.0	247	86

a Determined by SEC in THF using poly(methyl methacrylate) standards.

b Determined by TGA at 5% weight loss under N_2_.

c Determined by DSC.

**Figure 6:**
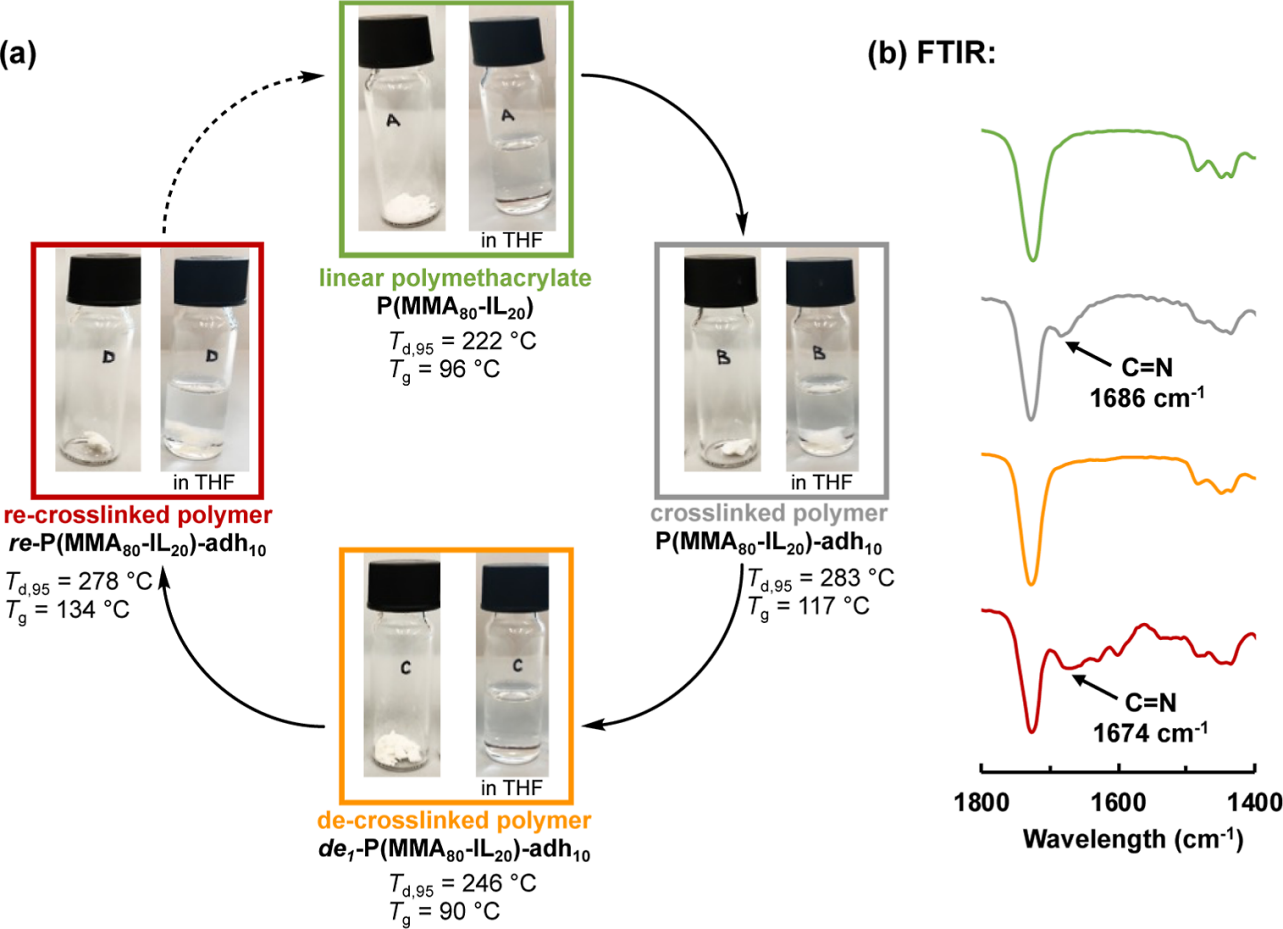
Crosslinking and de-crosslinking cycle of polymers in this work (a) and FTIR spectra of the resulting polymer samples showing the repeated appearance and disappearance of the C=N crosslinks (b). From the images, it can be seen that the crosslinked networks do not dissolve in THF.

After the de-crosslinking reaction was finished, the crude mixture was poured into a 5:1 (v/v) mixture of Et_2_O and isopropanol. The solid precipitate was filtered off and dried in a vacuum oven at 40 °C to obtain a white polymer powder. Besides the ^1^H NMR spectra recorded to monitor the de-crosslinking process, FTIR data of the dried polymers were collected that also indicated the absence of the C=N bond in the de-crosslinked polymers (Figures 6b and S22).

Analogously to the linear polymethacrylates, SEC analyses in THF were carried out for the de-crosslinked polymers (Table 3, Figure S26). The *M*_n_ of de-crosslinked polymers (Table 3, entries 1, 2, and 5) were very similar to the primary linear **P(MMA**_**80**_**-IL**_**20**_**)** (36.8 kg mol^–1^, D̵ = 1.6, Table 1, entry 4) that was used for the crosslinking experiments with **adh** or **mdh**. In addition, the SEC results of the primary non-crosslinked **P(MMA**_**40**_**-IL**_**60**_**)** (*M*_n_ = 20.4 kg mol^–1^, D̵ = 1.7, Table 1, entry 6) were also comparable to those of the corresponding de-crosslinked polymers (Table 3, entries 3, 4, and 6).

The *T*_d,95_ and *T*_g_ values (Table 3, and Figures S31 and S36) of the de-crosslinked polymers were generally higher than the respective values of the linear copolymers used for the crosslinking experiments. This may be because the dihydrazide unit was only partially removed in some cases. For example, only one acylhydrazone bond of the dihydrazide may have been cleaved from the levulinic unit during the de-crosslinking, leaving the dihydrazide still connected to the polymer. These residual units in the polymer chain would most probably not affect solubility properties (Table S1) and not the *M*_n_ values obtained by SEC, but they may still have a minor influence on the TGA and DSC data. Because the amount of dihydrazide is very small, these partially cleaved units are difficult to detect by ^1^H NMR (Figures S12–S17) and FTIR (Figure S22) spectroscopy. Based on the molecular weights, thermal data, and NMR and FTIR spectra, the de-crosslinking reactions of the crosslinked polymer networks under acidic conditions were regarded as successful.

### Re-crosslinking of a De-crosslinked Polymer with Adipic Dihydrazide

Finally, we demonstrated that a re-crosslinking of linear copolymers obtained from a de-crosslinked polymer network can be carried out repeatedly. We used the de-crosslinked polymer ***de***_***1***_-**P(MMA**_**80**_**-IL**_**20**_**)-adh**_**10**_ and performed a second crosslinking with **adh** using our standard conditions [GVL/H_2_O, 4:1 (v/v), cat. acetic acid Scheme 2]. The resulting re-crosslinked polymer, designated ***re*-P(MMA**_**80**_**-IL**_**20**_**)-adh**_**10**_, exhibited a *T*_d,95_ of 278 °C (Figure S32a), which was comparable to the *T*_d,95_ of the previously crosslinked polymethacrylate **P(MMA**_**80**_**-IL**_**20**_**)-adh**_**10**_ (283 °C, Table 2, entry 7). The *T*_g_ of the re-crosslinked polymer was 134 °C (Figure S37), which was even a bit higher than the *T*_g_ of the **P(MMA**_**80**_**-IL**_**20**_**)-adh**_**10**_ (117 °C). Additionally, a comparison of the FTIR spectra of the linear **P(MMA**_**80**_**-IL**_**20**_**)**, the crosslinked **P(MMA**_**80**_**-IL**_**20**_**)-adh**_**10**_, the de-crosslinked ***de***_***1***_-**P(MMA**_**80**_**-IL**_**20**_**)-adh**_**10**_, and the re-crosslinked ***re*-P(MMA**_**80**_**-IL**_**20**_**)-adh**_**10**_ polymers indicated the corresponding absence, formation, disappearance, and re-formation, respectively, of the C=N bond (Figure 6b). These experiments clearly illustrated that the sequential crosslinking/de-crosslinking using dihydrazides as the crosslinking agent can be carried out repeatedly with the same copolymer containing levulinoyl side chains (Figure 6a). This enables a route for chemical recycling of the corresponding network polymers.

## Conclusions

A highly regioselective two-step biocatalytic pathway for the synthesis of levulinic isosorbide methacrylate was developed in high yields using a simple chromatography-free workup. The addition of the levulinoyl group to the previously prepared 5-methacrylate isosorbide was performed using vinyl levulinate as the acyl donor in the presence of Novozym 435. This method provided the desired levulinic monomethacrylate of isosorbide in a high yield. Thereafter, a series of random copolymers of the levulinic isosorbide monomer and methyl methacrylate with different ratios were prepared and characterized. The thermal stability of the copolymers was high, and the *T*_g_ decreased with increase in the levulinic isosorbide content.

The levulinic ketone group in the side chains of the copolymers enabled the formation of crosslinked networks through acylhydrazone bonds obtained in reactions with adipic or malonic dihydrazides. These covalent adaptable networks exhibited higher thermal stability in comparison to the corresponding linear polymethacrylates. Additionally, the *T*_g_ also increased significantly with adipic dihydrazide and even higher with malonic dihydrazide as the crosslinking agent. The cleavage of the dynamic acylhydrazone linkages under acidic conditions was demonstrated, and the linear polymethacrylates were recovered. A successful second crosslinking experiment with a de-crosslinked polymer and adipic dihydrazide was carried out to illustrate the repetitive crosslinking/de-crosslinking feature of the polymers functionalized with levulinic side chains. The sequential C=N bond formation/cleavage in the acylhydrazone linkages was conveniently observed by FTIR analysis. Furthermore, the solubility of the linear polymethacrylates and insolubility of the corresponding crosslinked networks in common organic solvents (CHCl_3_, THF, ACN, DMSO) further confirmed the presence of the dynamic acylhydrazone bonds in these polymers. Consequently, the novel levulinic isosorbide monomethacrylate monomer provides an opportunity to synthesize biobased polymers with a reversible crosslinking feature when reacted with dihydrazides. Such a strategy of biobased covalent adaptable networks is highly advantageous considering the recyclability and reusability of thermoset polymers.

## Supporting Information Available

Characterization data of vinyl levulinate and polymers, ^1^H and ^13^C NMR spectra, FTIR spectra, SEC, TGA, and DTG profiles, DSC curves, and solubility of polymers (PDF)
