# Supplementary material for: Covalent Adaptable Polymethacrylate Networks by Hydrazide Crosslinking Via Isosorbide Levulinate Side Groups
Source: ACS Sustain Chem Eng. 2023 May 19;11(22):8294–307. doi: 10.1021/acssuschemeng.3c00747 (PMC10245394; doi:10.1021/acssuschemeng.3c00747)
Supplement: Supplementary file 1 — sc3c00747_si_001.pdf [file sc3c00747_si_001.pdf]

Supporting Information

Covalent Adaptable Polymethacrylate Networks by Hydrazide Crosslinking via Isosorbide Levulinate Side Groups

Livia Matt,[a] Rauno Sedrik,[a] Olivier Bonjour,[b] Miglé Vasiliaukaité,[a] Patric Jannasch,*[a,b] and Lauri Vares*[a]

[a] Institute of Technology, University of Tartu, Nooruse 1, Tartu 50411, Estonia
[b] Department of Chemistry, Lund University, Box 124, Lund 221 00, Sweden

*E-mail: patric.jannasch@chem.lu.se, lauri.vares@ut.ee

Number of Pages: 25
Number of Figures: 37
Number of Tables: 1

Table of Contents

1. Characterization data S3
Vinyl levulinate S3
Linear polymethacrylates S3
Crosslinked polymers S3
De-crosslinked polymers S4
2. ^{1}H and ^{13}C NMR Spectra S5
Figure S1. ^{1}H and ^{13}C NMR spectra of IL. S5
Figure S2. Crude ^{1}H NMR spectrum of IL synthesis in MTBE after enzyme filtration and drying under reduced pressure (purity of the obtained IL >95%). S6
Figure S3. Crude ^{1}H NMR spectrum of IL synthesis in ACN after enzyme filtration and drying under reduced pressure (purity of the obtained IL >95%). S6
Figure S4. ^{1}H NMR spectrum of PMMA synthesized using the same procedure as for other (co)polymethacrylates in this study. S7
Figure S5. ^{1}H NMR spectrum of PIL. S7
Figure S6. ^{1}H NMR spectrum of linear copolymer P(MMA$_{95}$-IL$_{5}$). S8
Figure S7. ^{1}H NMR spectrum of linear copolymer P(MMA$_{90}$-IL$_{10}$). S8
Figure S8. ^{1}H NMR spectrum of linear copolymer P(MMA$_{80}$-IL$_{20}$). S9
Figure S9. ^{1}H NMR spectrum of linear copolymer P(MMA$_{60}$-IL$_{40}$). S9
Figure S10. ^{1}H NMR spectrum of linear copolymer P(MMA$_{40}$-IL$_{60}$). S10
Figure S11. ^{1}H NMR spectrum of linear copolymer P(MMA$_{20}$-IL$_{80}$). S10

Figure S12. ^{1}H NMR spectrum of de-crosslinked polymer *de₁*-P(MMA$_{80}$-IL$_{20}$)-adh$_{10}$. S11
Figure S13. ^{1}H NMR spectrum of de-crosslinked polymer *de₄*-P(MMA$_{80}$-IL$_{20}$)-adh$_{10}$. S11
Figure S14. ^{1}H NMR spectrum of de-crosslinked polymer *de₁*-P(MMA$_{40}$-IL$_{60}$)-adh$_{30}$. S12
Figure S15. ^{1}H NMR spectrum of de-crosslinked polymer *de₄*-P(MMA$_{40}$-IL$_{60}$)-adh$_{30}$. S12
Figure S16. ^{1}H NMR spectrum of de-crosslinked polymer *de₁*-P(MMA$_{80}$-IL$_{20}$)-mdh$_{10}$. S13
Figure S17. ^{1}H NMR spectrum of de-crosslinked polymer *de₄*-P(MMA$_{40}$-IL$_{60}$)-mdh$_{30}$. S13

3. IR Spectra S14

Figure S18. FTIR curves of linear (co)polymethacrylates. S14
Figure S19. FTIR curves of attempted adh- and mdh-crosslinking experiments with PMMA. S14
Figure S20. FTIR curves of adh-crosslinked polymers. S15
Figure S21. FTIR curves of mdh-crosslinked polymers. S16
Figure S22. FTIR curves of de-crosslinked polymers. S16
Figure S23. FTIR curve of re-crosslinked polymer. S17

4. SEC Graphs S18

Figure S24. SEC curves in THF of linear (co)polymethacrylates (data shown in two plots for clarity). S18
Figure S25. SEC curves in THF of attempted adh- and mdh-crosslinking experiments with PMMA. S18
Figure S26. SEC curves in THF of de-crosslinked polymers (data shown in two plots for clarity). S18

5. TGA and DTG Graphs S19

Figure S27. TGA (a) and DTG (b) curves of linear (co)polymethacrylates. S19
Figure S28. TGA curves of adh-crosslinked polymers (data shown in two plots for clarity). S19
Figure S29. DTG curves of adh-crosslinked polymers (data shown in two plots for clarity). S20
Figure S30. TGA (a) and DTG (b) curves of mdh-crosslinked polymers. S20
Figure S31. TGA (a) and DTG (b) curves of de-crosslinked polymers. S21
Figure S32. TGA (a) curve and DTG (b) curve of re-crosslinked polymer. S21

6. DSC Graphs S22

Figure S33. DSC second heating scans for linear (co)polymethacrylates. S22
Figure S34. DSC second heating scans for adh-crosslinked polymers. S22
Figure S35. DSC second heating scans for mdh-crosslinked polymers. S23
Figure S36. DSC second heating scans for de-crosslinked polymers. S23
Figure S37. DSC second heating scan for re-crosslinked polymer. S23

7. Solubility S24

Table S1. Solubility of linear polymethacrylates, crosslinked polymers, de-crosslinked polymers, and a re-crosslinked polymer at 21 °C. S24

References S25

1. CHARACTERIZATION DATA

Vinyl levulinate

^{1}H NMR (400.1 MHz, $CDCl_3$) δ 7.21 (dd, J = 14.0, 6.3 Hz, 1H), 4.86 (dd, J = 14.0, 1.6 Hz, 1H), 4.55 (dd, J = 6.3, 1.6 Hz, 1H), 2.76 (t, J = 6.7 Hz, 2H), 2.63 (t, J = 6.7 Hz, 2H), 2.18 (s, 3H) ppm. ^{13}C NMR (100.6 MHz, $CDCl_3$) δ 206.14, 169.93, 141.08, 97.78, 37.55, 29.76, 27.62 ppm. The NMR data is consistent with the previous literature report.[1]

Linear polymethacrylates

PMMA: IR (ATR) ν_{max} (cm^{-1}): 2951, 1724, 1451, 1389, 1246, 729. ^{1}H NMR (400.1 MHz, $CDCl_3$) δ 3.59 (bs, 3H), 2.09–1.33 (m, 2H), 1.30–0.71 (m, 3H) ppm.

PIL: IR (ATR) ν_{max} (cm^{-1}): 2967, 1721, 1485, 1366, 1204, 1153, 729. ^{1}H NMR (400.1 MHz, $CDCl_3$) δ 5.16 (bs, 1H), 5.01 (bs, 1H), 4.73 (bs, 1H), 4.47 (bs, 1H), 4.12–3.59 (m, 4H), 2.76 (m, 2H), 2.56 (m, 2H), 2.18 (s, 3H), 2.09–1.40 (m, 2H), 1.37–0.71 (m, 3H) ppm.

General characterization data for linear copolymers **P(MMA$_x$-IL$_y$)**: IR (ATR) ν_{max} (cm^{-1}): 2951–2936, 1728–1721, 1485–1450, 1389–1366, 1242–1204, 1153–1150, 756–729. ^{1}H NMR (400.1 MHz, $CDCl_3$) δ 5.28–5.13 (bs, 1H), 5.12–4.91 (m, 1H), 4.85–4.64 (m, 1H), 4.57–4.38 (m, 1H), 4.10–3.64 (m, 4H), 3.63–3.36 (m, 3H), 2.87–2.68 (m, 2H), 2.67–2.45 (m, 2H), 2.29–2.11 (m, 3H), 2.10–1.33 (m, 4H), 1.32–0.57 (m, 6H) ppm.

Crosslinked polymers

PIL-adh$_5$: IR (ATR) ν_{max} (cm^{-1}): 2936, 1732, 1686, 1420, 1366, 1207, 1150, 764.

General characterization data for **adh**-crosslinked copolymers of **MMA** and **IL** (**PMMA$_x$-IL$_y$)-adh$_z$**): IR (ATR) ν_{max} (cm^{-1}): 2951–2936, 1732–1721, 1686–1674, 1435–1419, 1389–1362, 1207–1188, 1150–1146, 764–752.

PIL-mdh$_5$: IR (ATR) ν_{max} (cm^{-1}): 2947, 1732, 1686, 1420, 1366, 1207, 1150, 764.

General characterization data for **mdh**-crosslinked copolymers of **MMA** and **IL** (**PMMA$_x$-IL$_y$)-mdh$_z$**): IR (ATR) ν_{max} (cm^{-1}): 2951–2943, 1732–1728, 1693–1686, 1435–1420, 1389–1366, 1207–1188, 1150–1146, 764–748.

***re*-P(MMA$_{80}$-IL$_{20}$)-adh$_{10}$**: IR (ATR) ν_{max} (cm^{-1}): 2951, 1728, 1674, 1435, 1389, 1188, 1146, 752.

De-crosslinked polymers

The ^{1}H NMR and FTIR data of the de-crosslinked copolymers were consistent with the corresponding linear polymethacrylates.

2. ^{1}H AND ^{13}C NMR SPECTRA

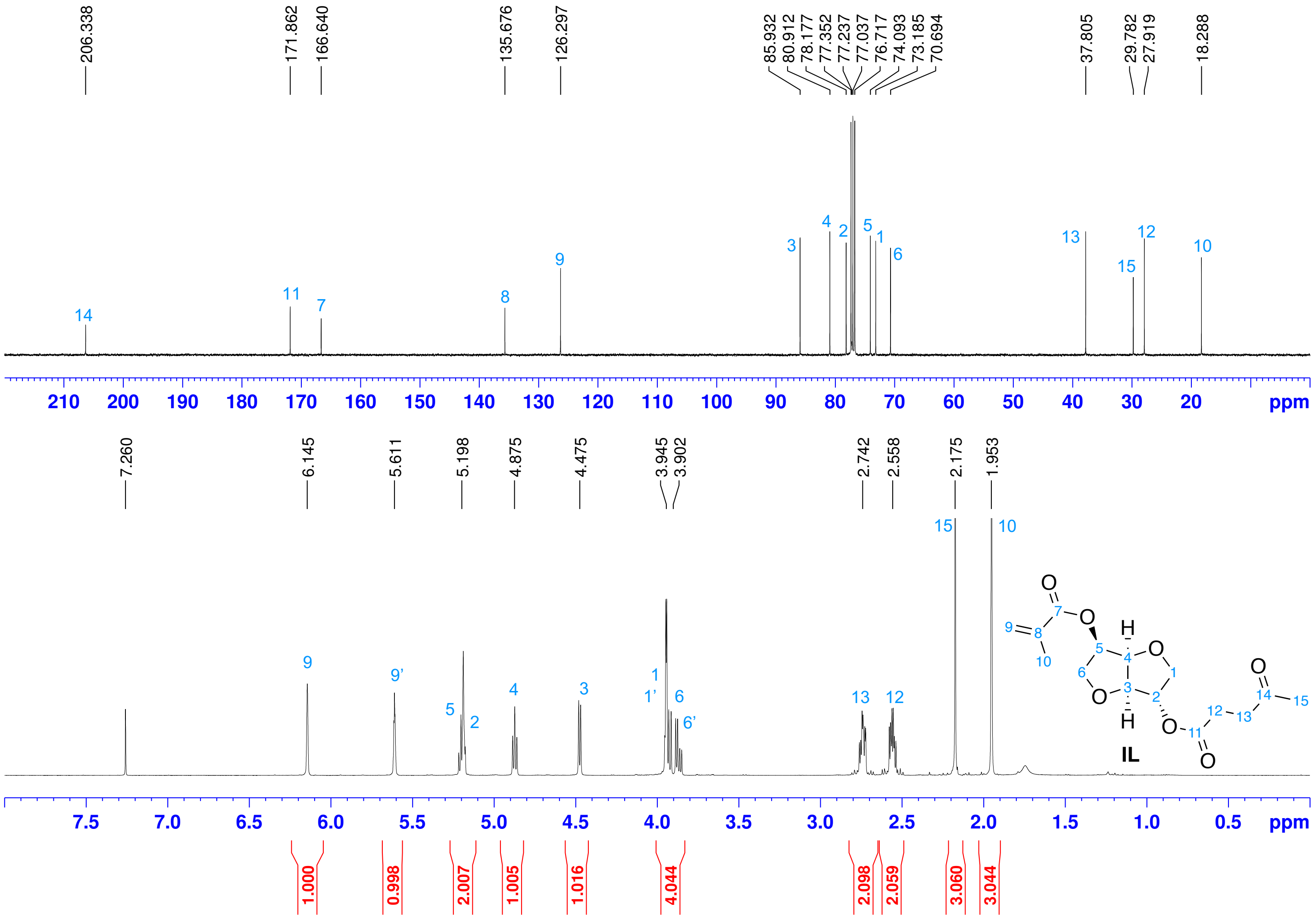

Figure S1. ^{1}H and ^{13}C NMR spectra of **IL**.

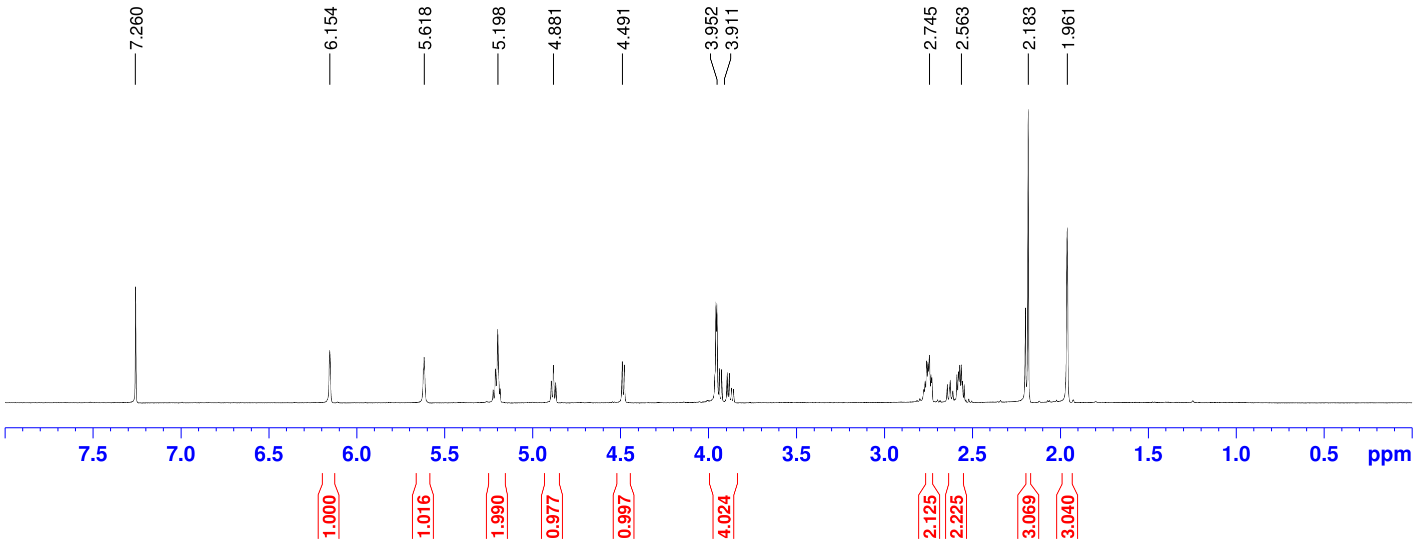

Figure S2. Crude ^{1}H NMR spectrum of **IL** synthesis in MTBE after enzyme filtration and drying under reduced pressure (purity of the obtained **IL** >95%).

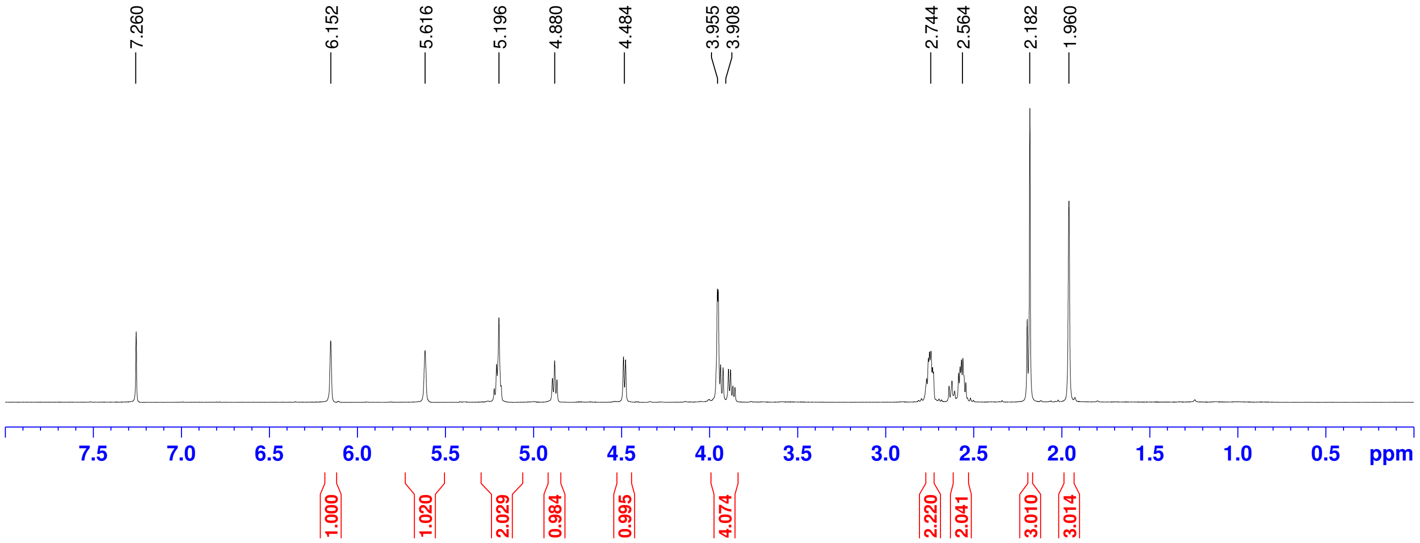

Figure S3. Crude ^{1}H NMR spectrum of **IL** synthesis in ACN after enzyme filtration and drying under reduced pressure (purity of the obtained **IL** >95%).

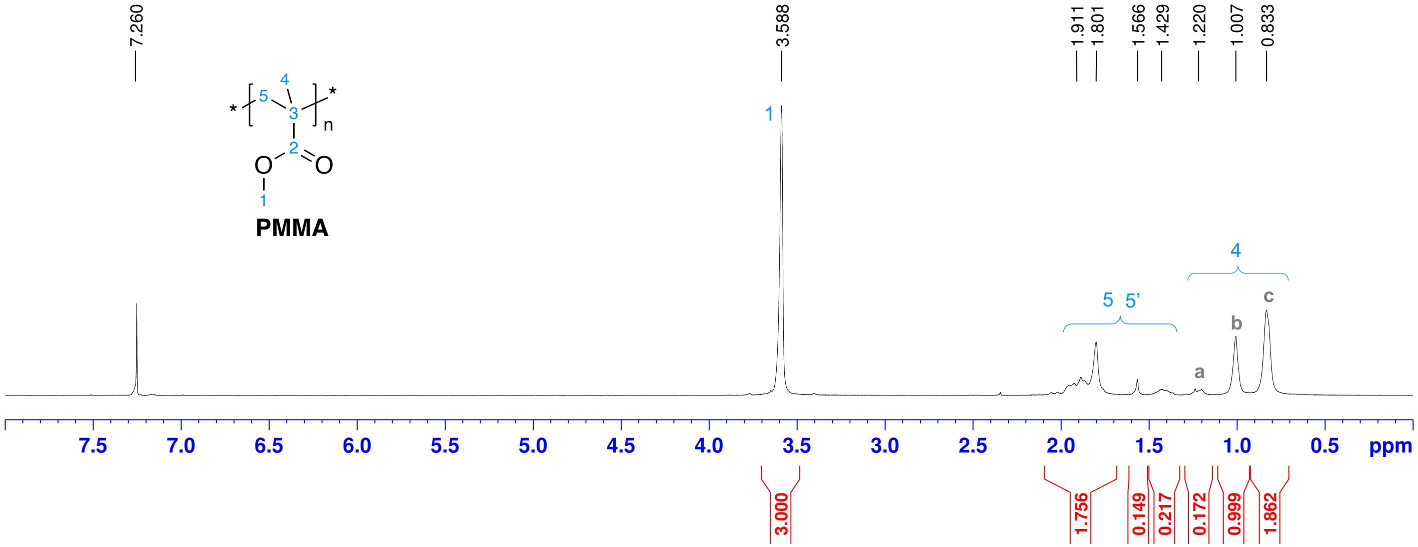

Figure S4. ^{1}H NMR spectrum of **PMMA** synthesized using the same procedure as for other (co)polymethacrylates in this study. Signals **a**, **b** and **c** were used to determine the *mm* (6%), *rm* (33%), and *rr* (61%) triad fractions, respectively.

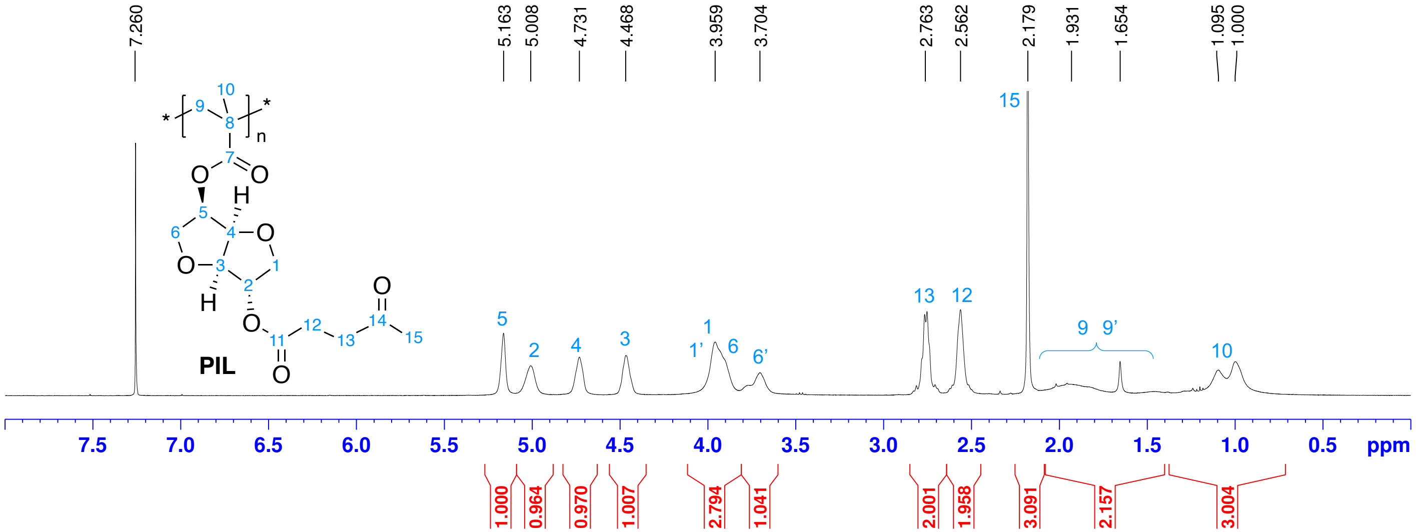

Figure S5. ^{1}H NMR spectrum of **PIL**.

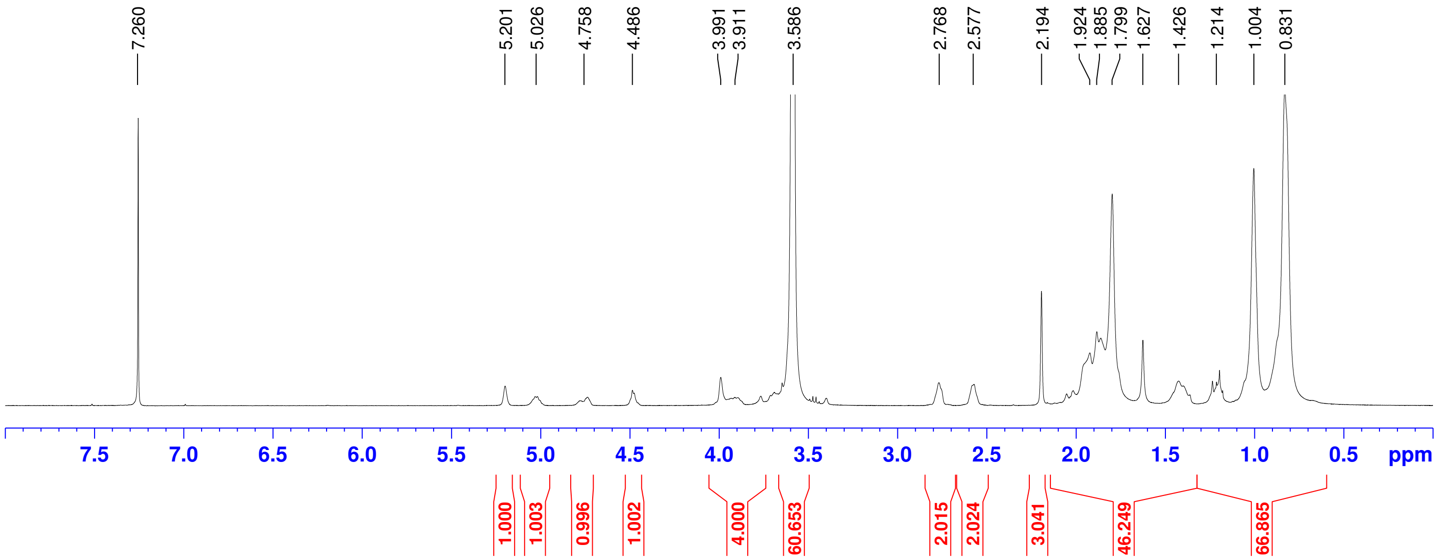

Figure S6. ^{1}H NMR spectrum of linear copolymer **P(MMA$_{95}$-IL$_5$)**.

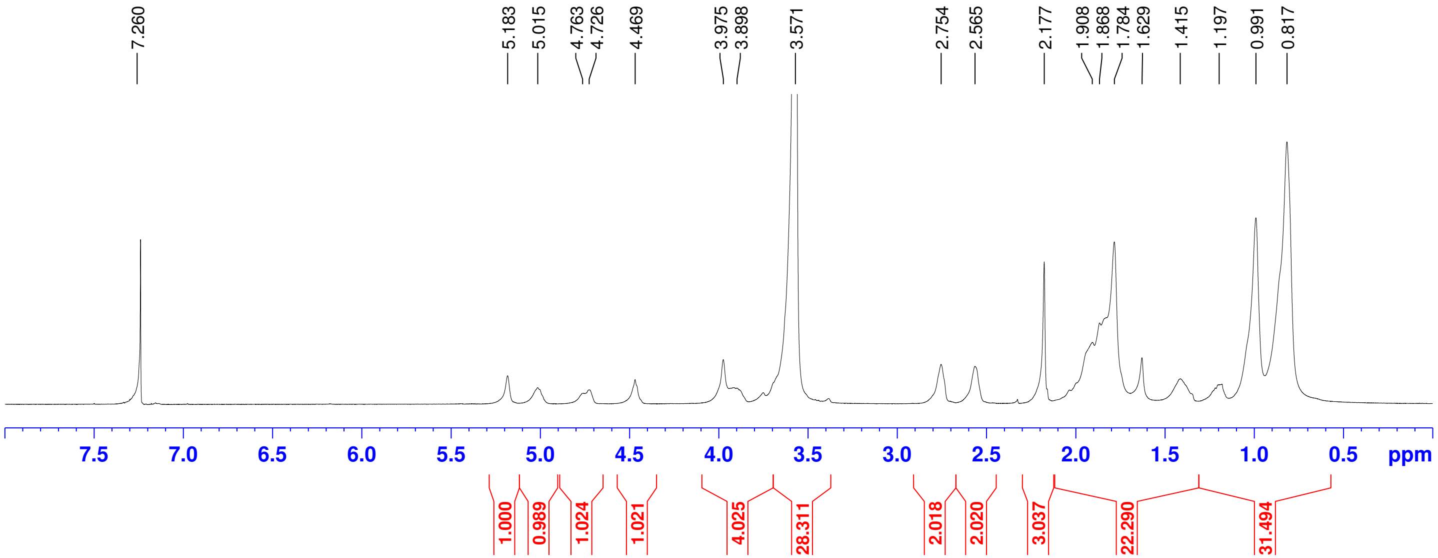

Figure S7. ^{1}H NMR spectrum of linear copolymer **P(MMA$_{90}$-IL$_{10}$)**.

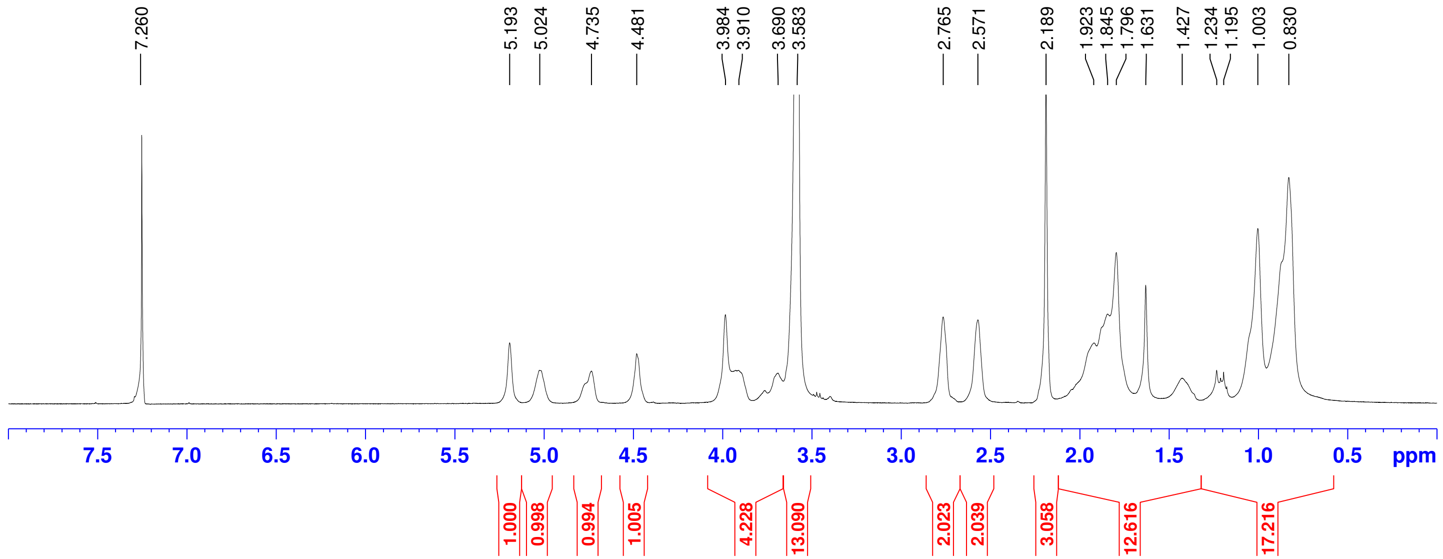

Figure S8. ^{1}H NMR spectrum of linear copolymer **P(MMA$_{80}$-IL$_{20}$)**.

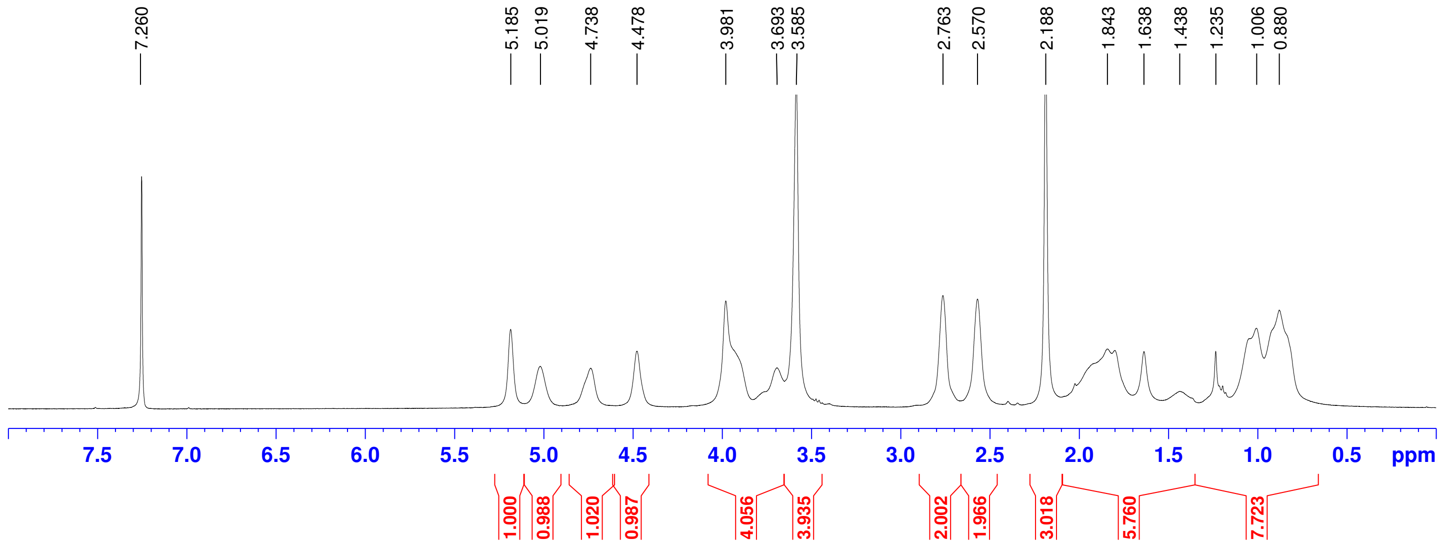

Figure S9. ^{1}H NMR spectrum of linear copolymer **P(MMA$_{60}$-IL$_{40}$)**.

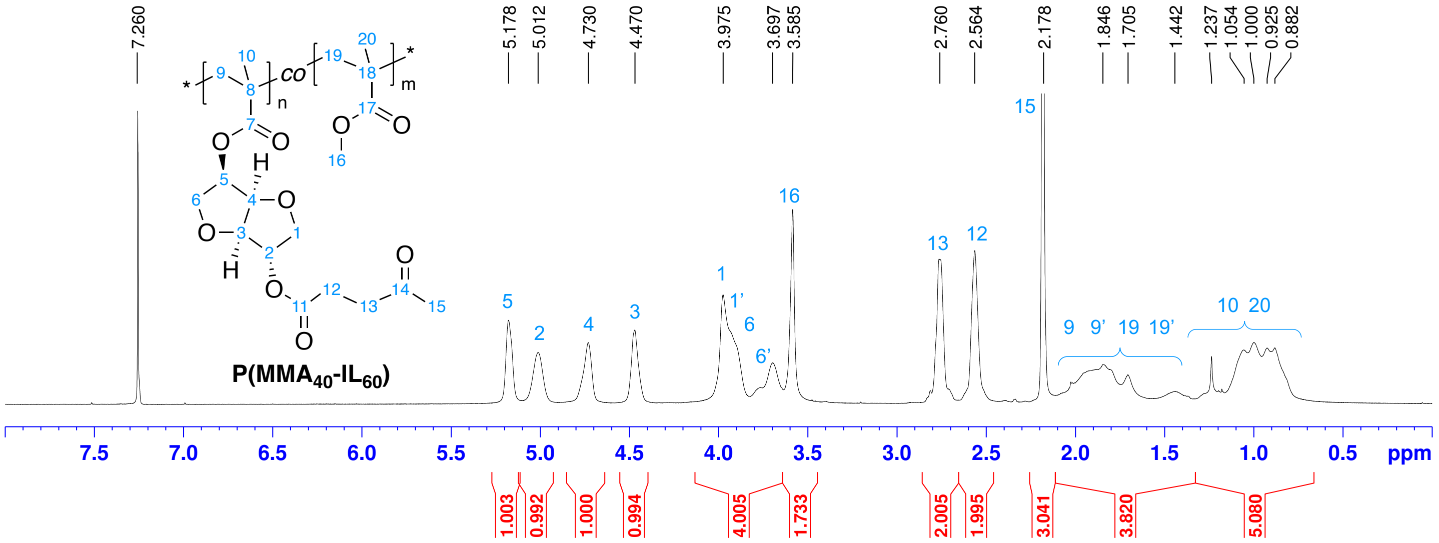

Figure S10. ^{1}H NMR spectrum of linear copolymer **P(MMA$_{40}$-IL$_{60}$)**.

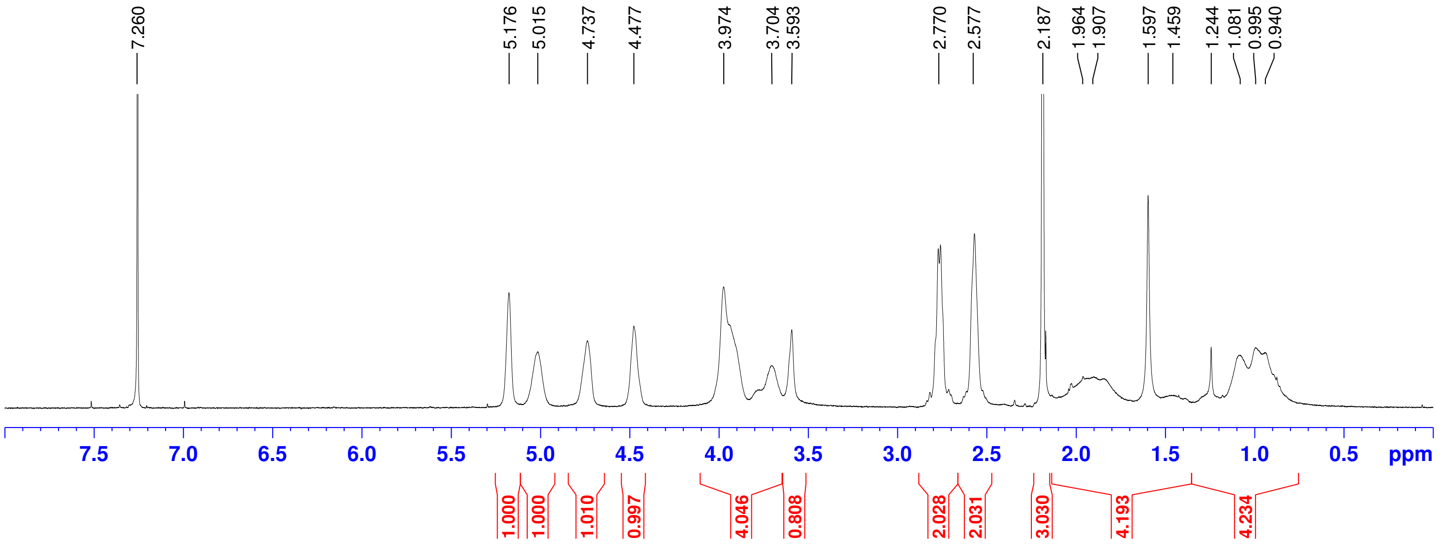

Figure S11. ^{1}H NMR spectrum of linear copolymer **P(MMA$_{20}$-IL$_{80}$)**.

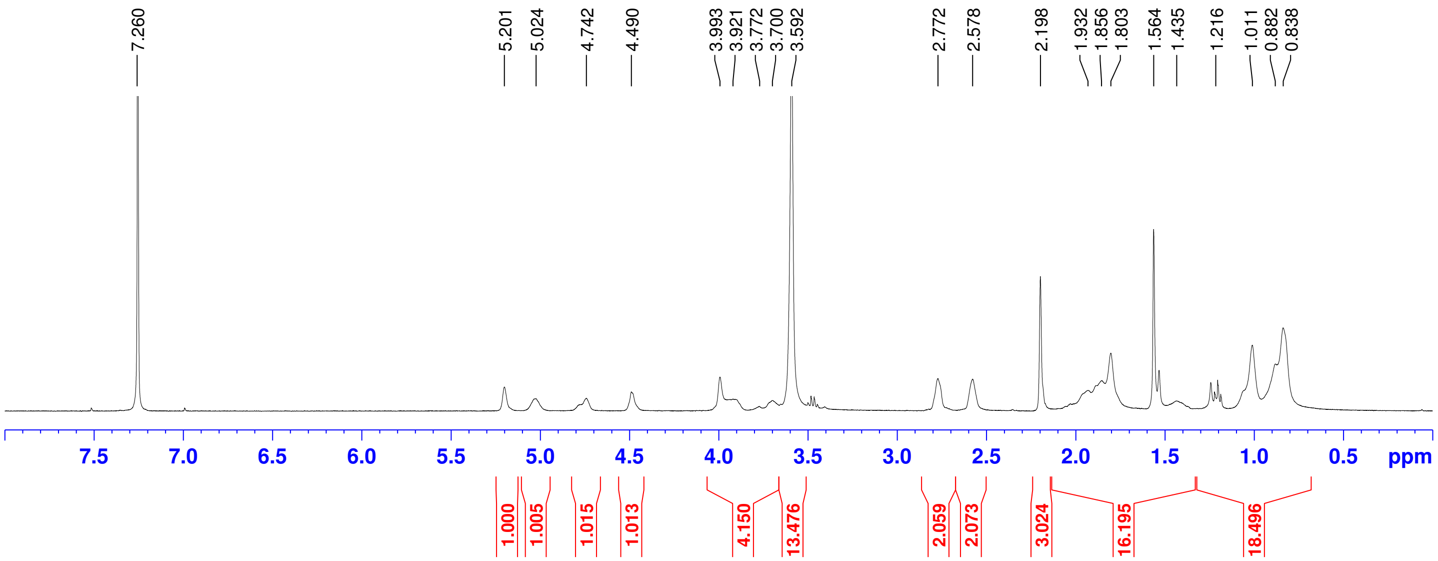

Figure S12. ^{1}H NMR spectrum of de-crosslinked polymer ***de$_1$*-P(MMA$_{80}$-IL$_{20}$)-adh$_{10}$**.

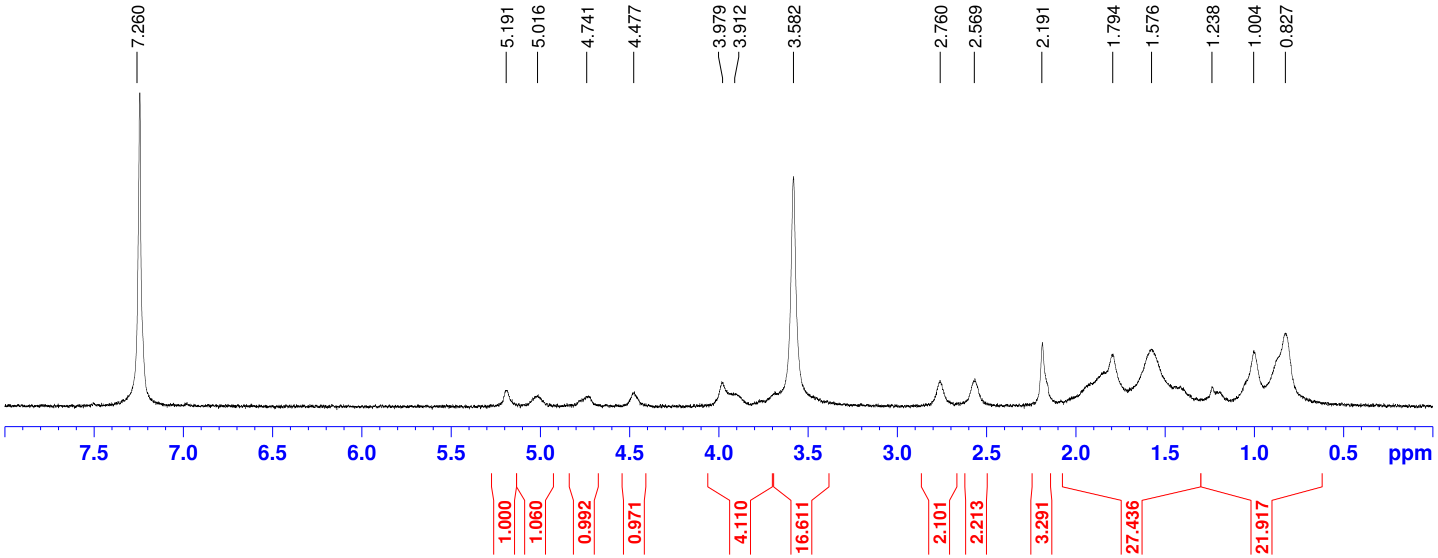

Figure S13. ^{1}H NMR spectrum of de-crosslinked polymer ***de$_4$*-P(MMA$_{80}$-IL$_{20}$)-adh$_{10}$**.

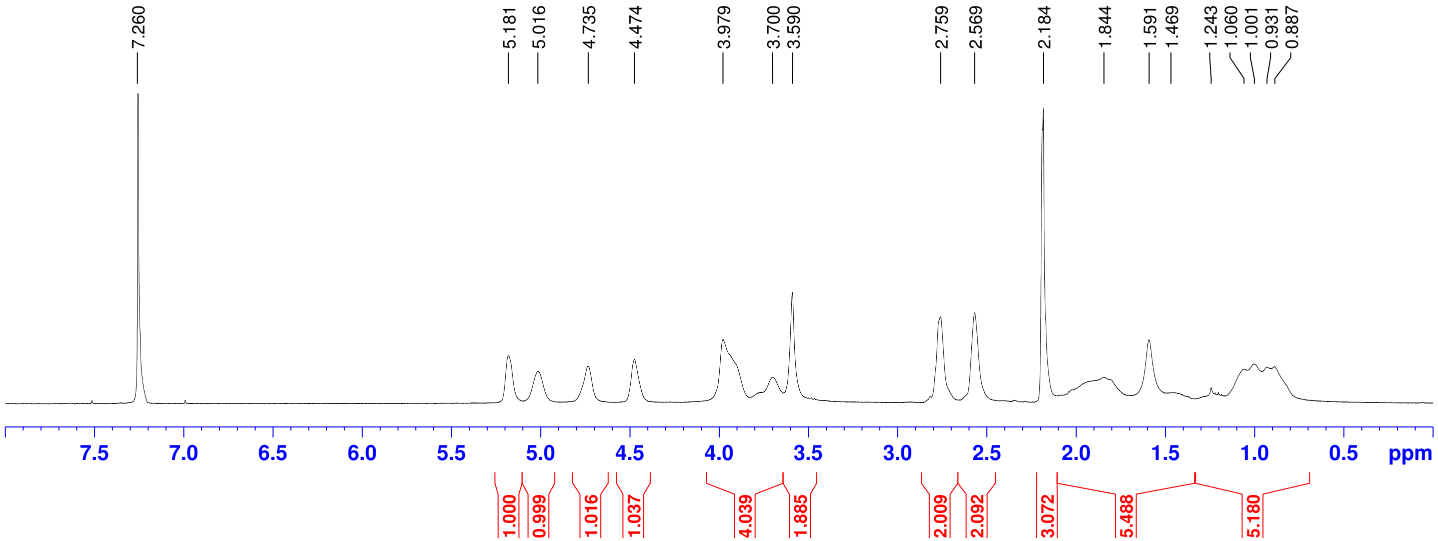

Figure S14. ^{1}H NMR spectrum of de-crosslinked polymer ***de₁*-P(MMA$_{40}$-IL$_{60}$)-adh$_{30}$**.

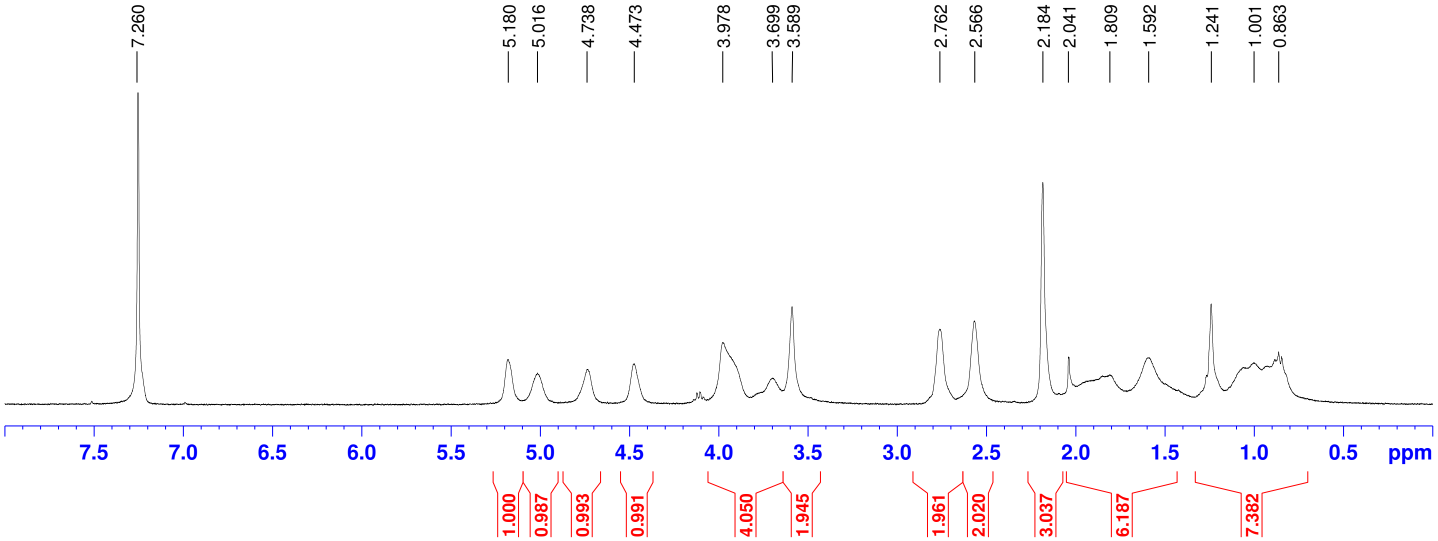

Figure S15. ^{1}H NMR spectrum of de-crosslinked polymer ***de$_4$*-P(MMA$_{40}$-IL$_{60}$)-adh$_{30}$**.

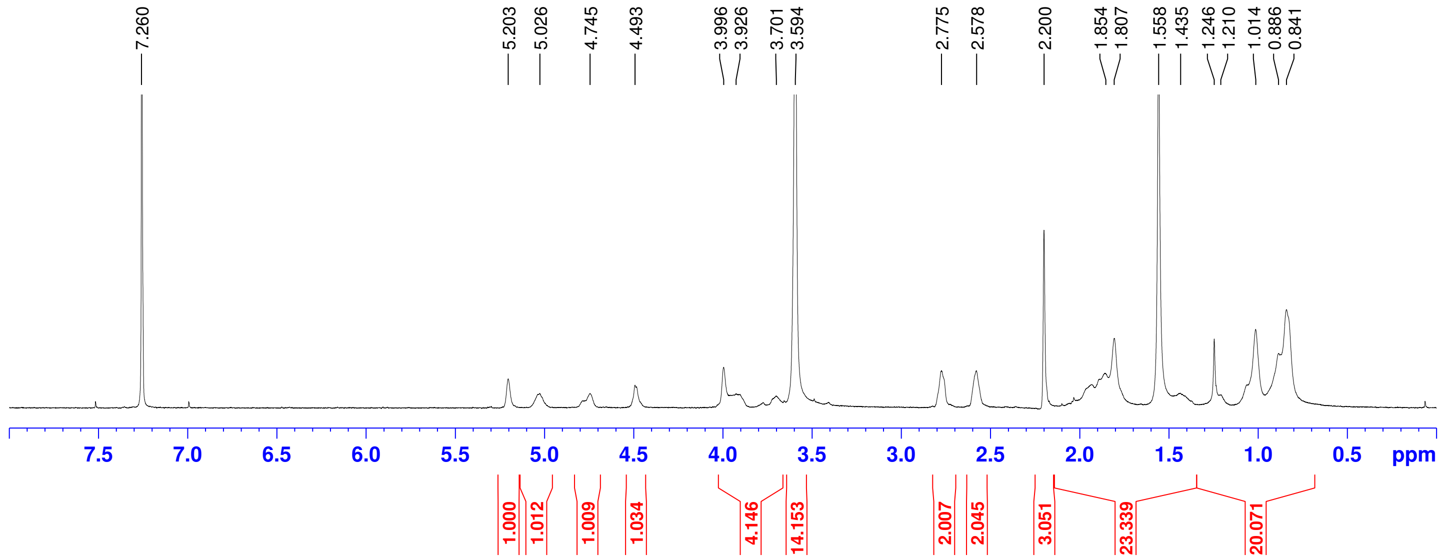

Figure S16. ^{1}H NMR spectrum of de-crosslinked polymer ***de1*-P(MMA80-IL20)-mdh10**.

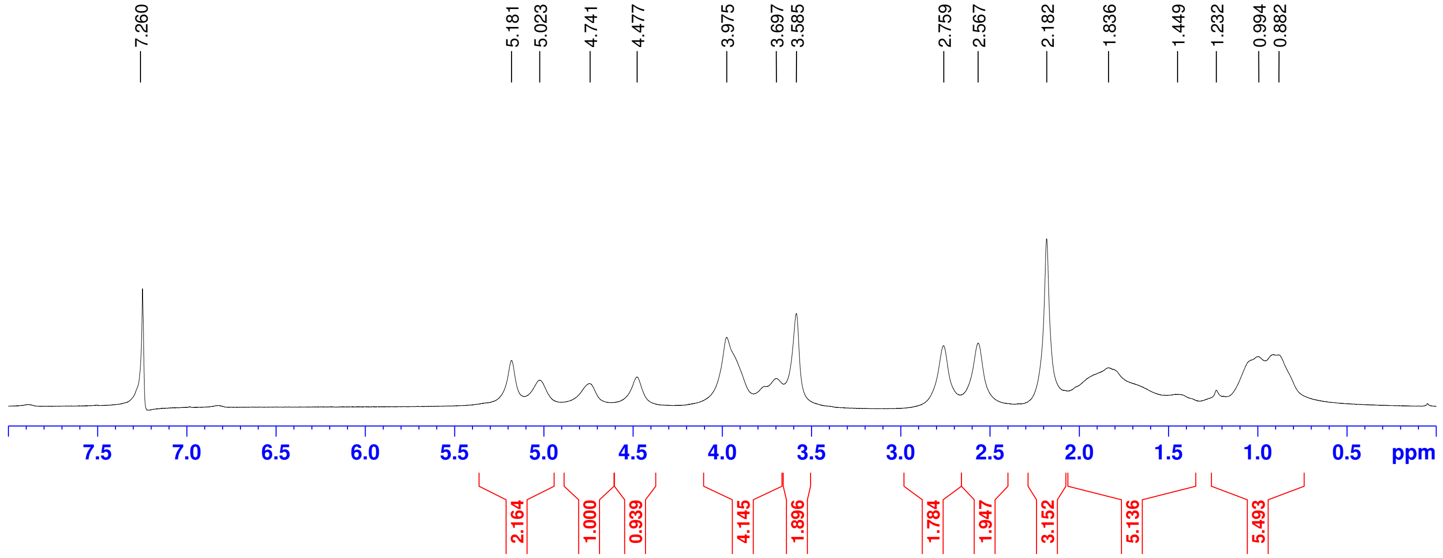

Figure S17. ^{1}H NMR spectrum of de-crosslinked polymer ***de4*-P(MMA40-IL60)-mdh30**.

3. IR SPECTRA

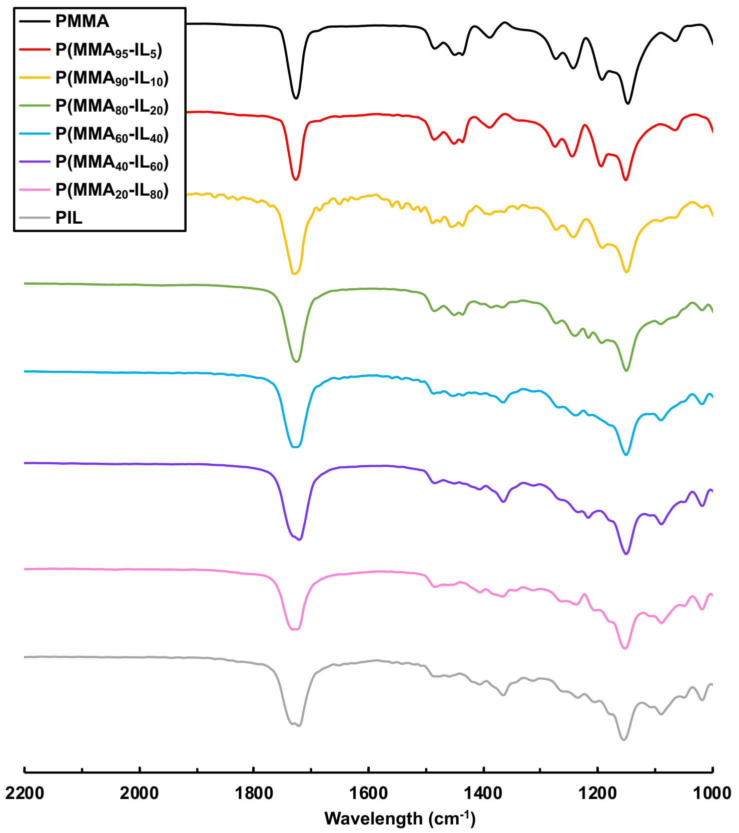

Figure S18. FTIR curves of linear (co)polymethacrylates.

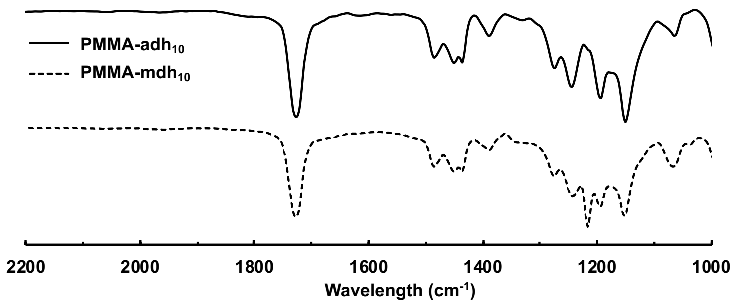

Figure S19. FTIR curves of attempted **adh**- and **mdh**-crosslinking experiments with **PMMA**.

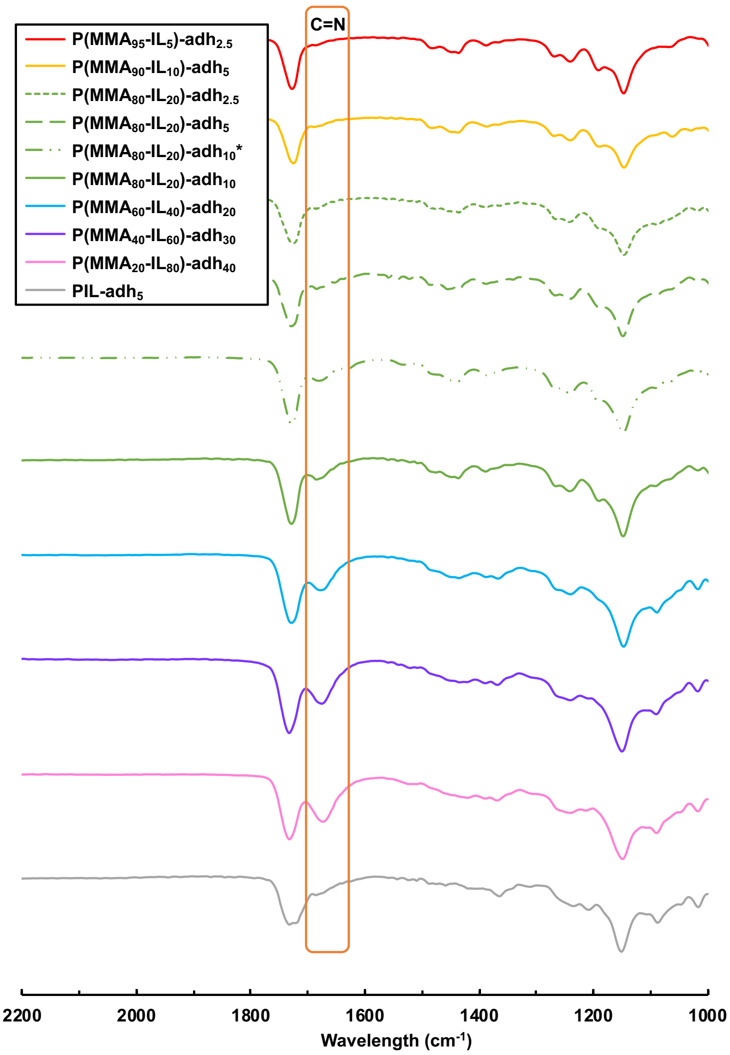

Figure S20. FTIR curves of **adh**-crosslinked polymers.

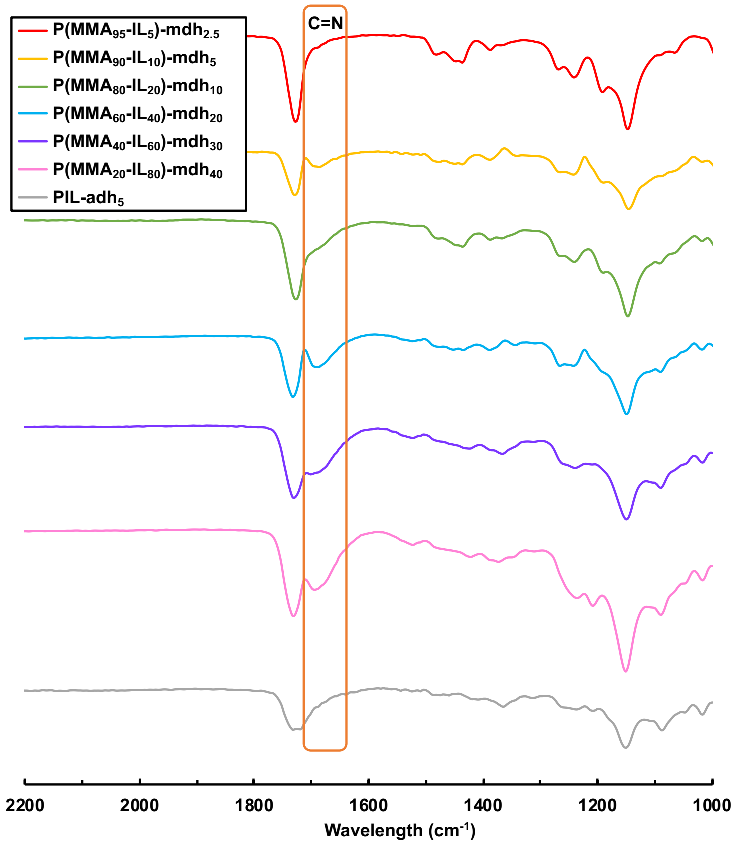

Figure S21. FTIR curves of **mdh**-crosslinked polymers.

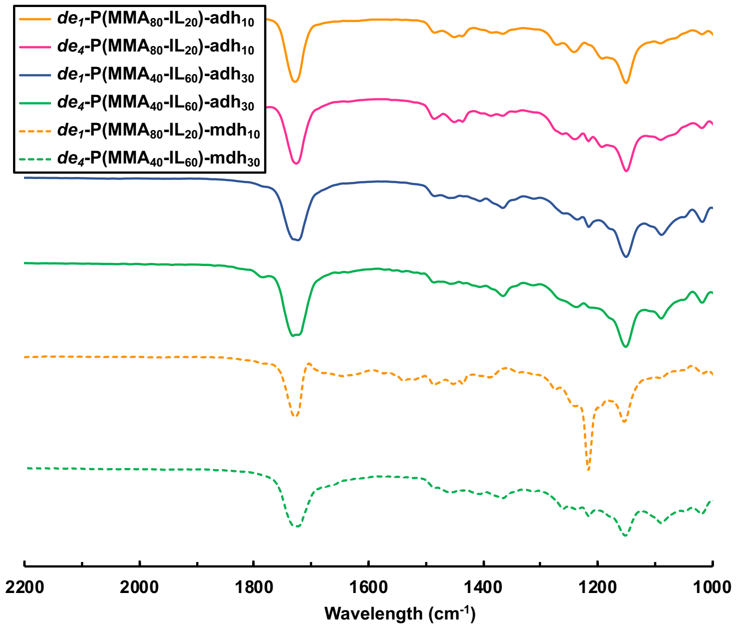

Figure S22. FTIR curves of de-crosslinked polymers.

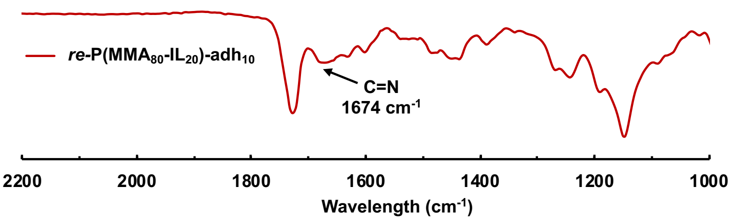

Figure S23. FTIR curve of re-crosslinked polymer.

4. SEC GRAPHS

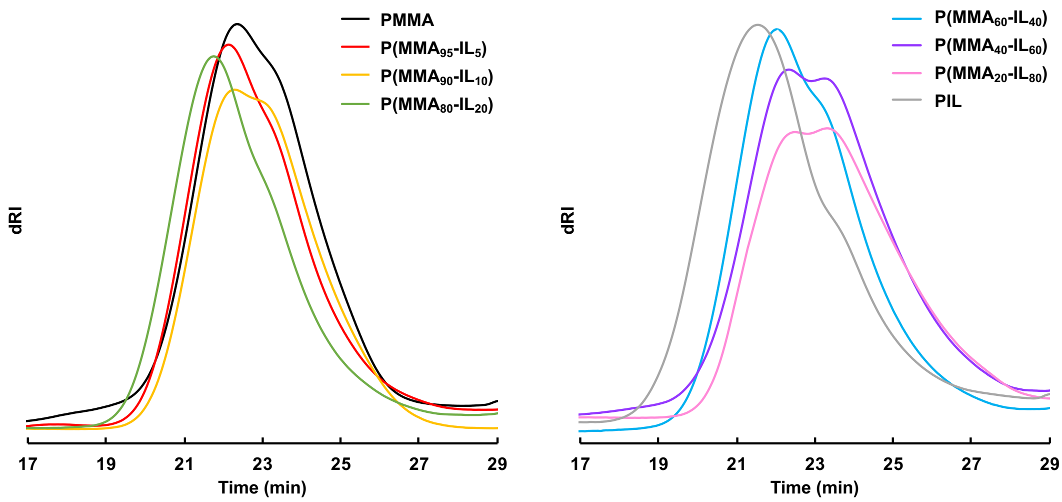

Figure S24. SEC curves in THF of linear (co)polymethacrylates (data shown in two plots for clarity).

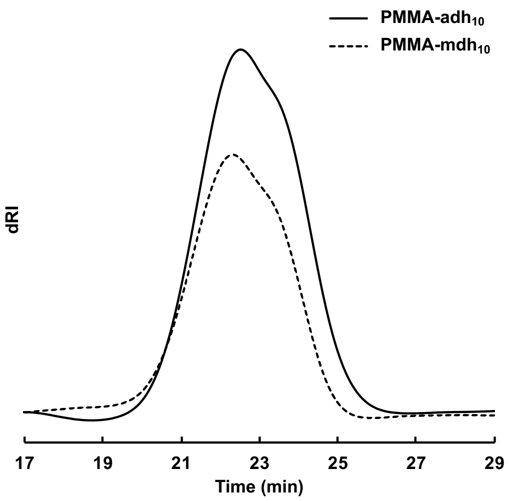

Figure S25. SEC curves in THF of attempted **adh**- and **mdh**-crosslinking experiments with **PMMA**.

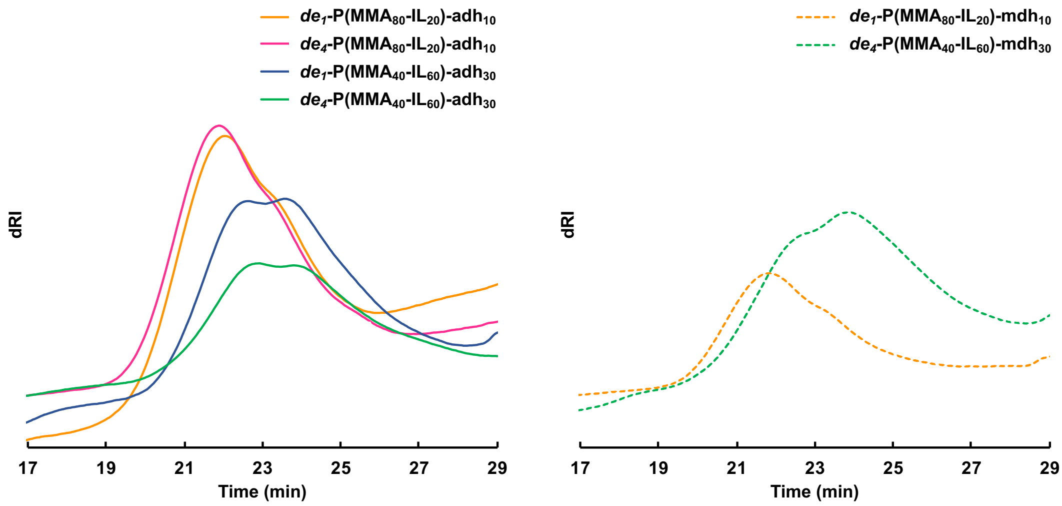

Figure S26. SEC curves in THF of de-crosslinked polymers (data shown in two plots for clarity).

5. TGA AND DTG GRAPHS

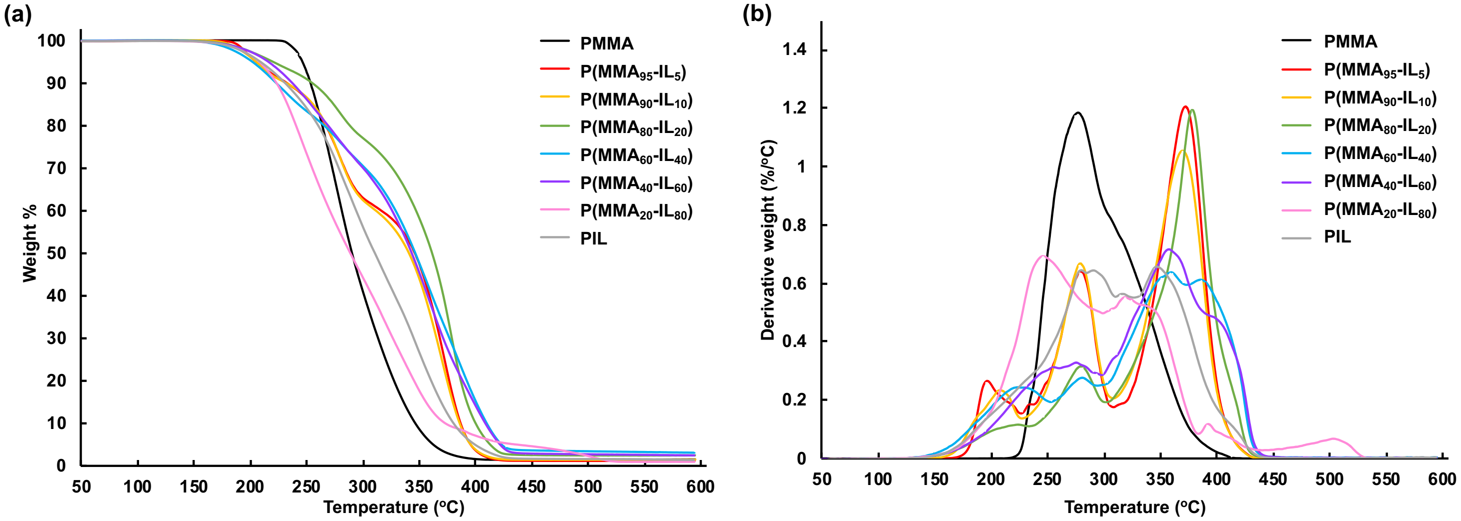

Figure S27. TGA (a) and DTG (b) curves of linear (co)polymethacrylates.

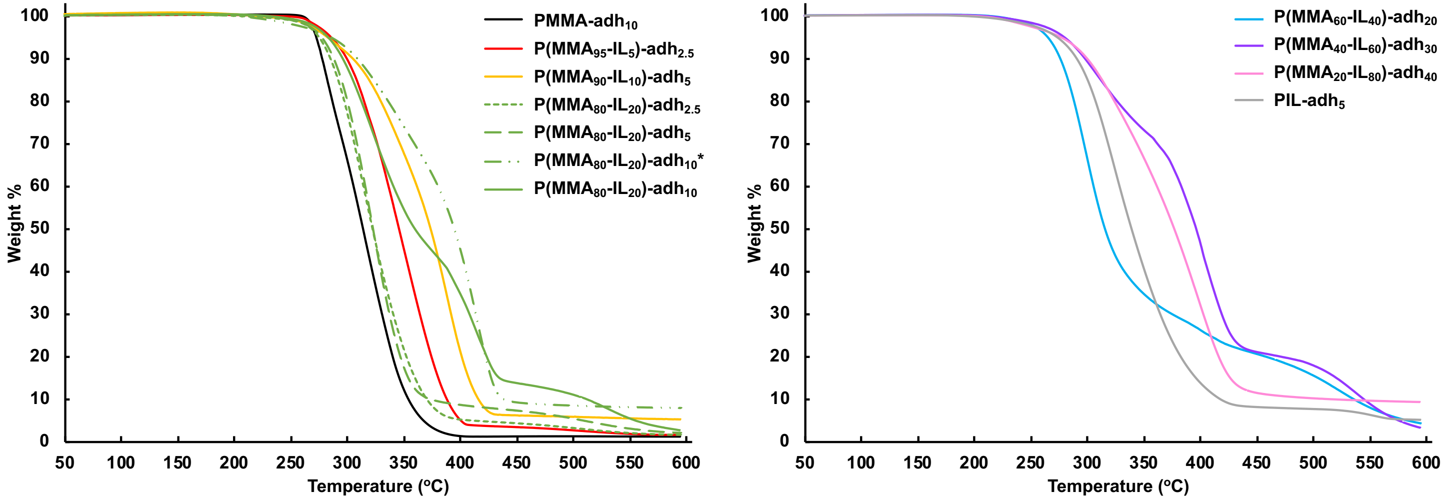

Figure S28. TGA curves of **adh**-crosslinked polymers (data shown in two plots for clarity).

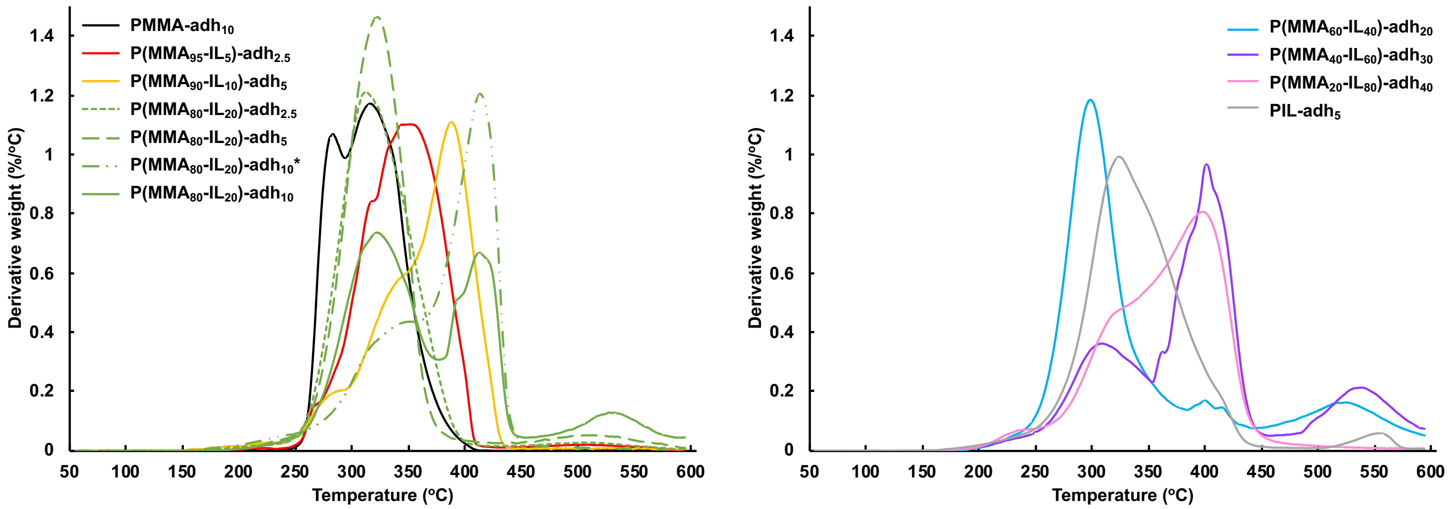

Figure S29. DTG curves of **adh**-crosslinked polymers (data shown in two plots for clarity).

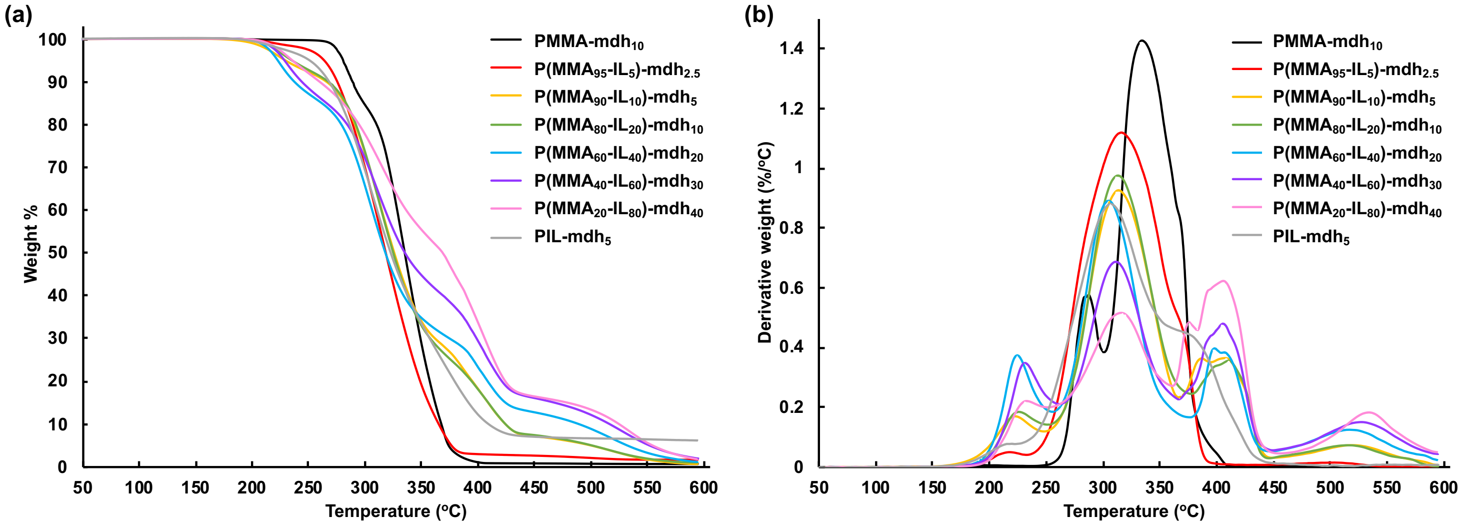

Figure S30. TGA (a) and DTG (b) curves of **mdh**-crosslinked polymers.

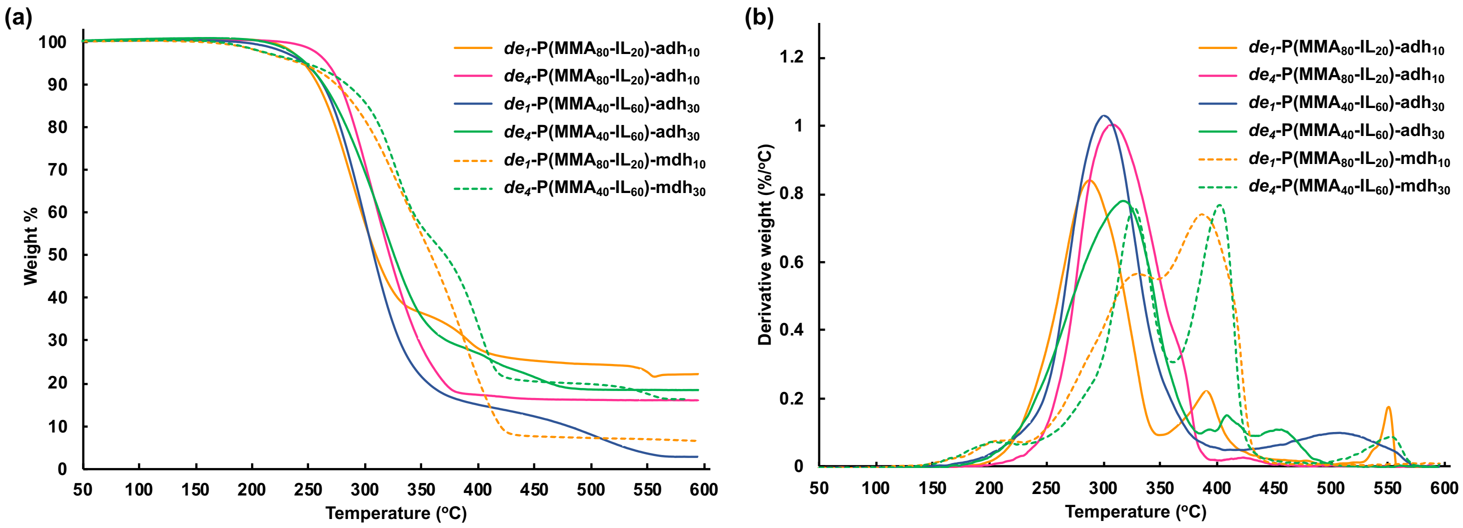

Figure S31. TGA (a) and DTG (b) curves of de-crosslinked polymers.

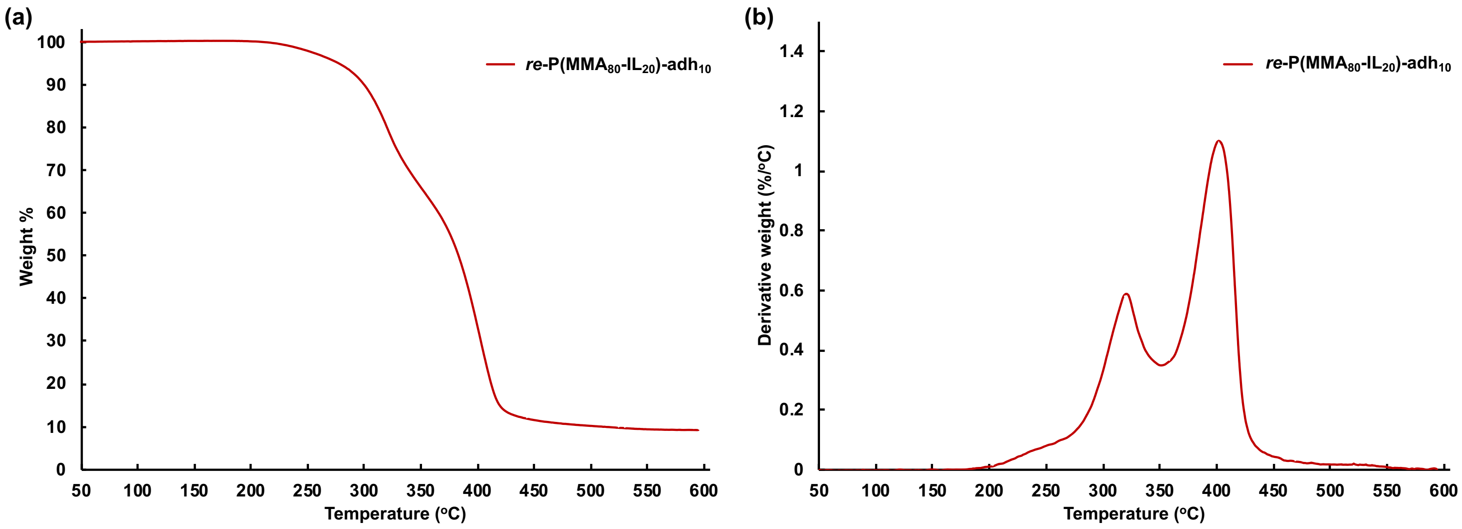

Figure S32. TGA (a) curve and DTG (b) curve of re-crosslinked polymer.

6. DSC GRAPHS

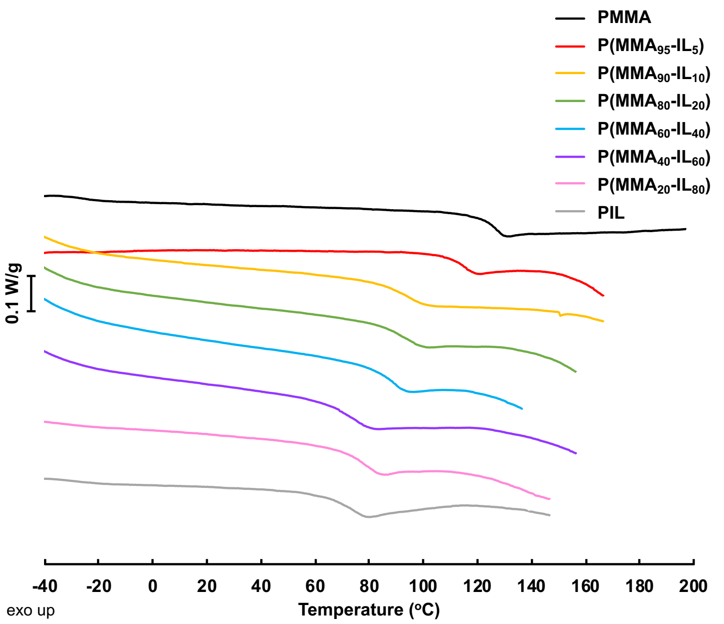

Figure S33. DSC second heating scans for linear (co)polymethacrylates.

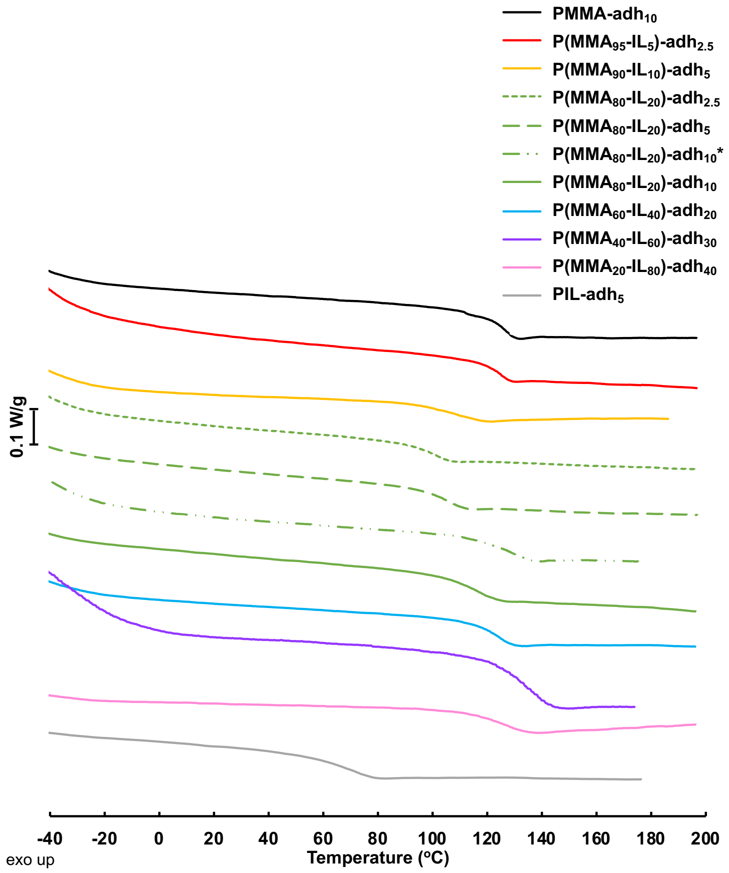

Figure S34. DSC second heating scans for **adh**-crosslinked polymers.

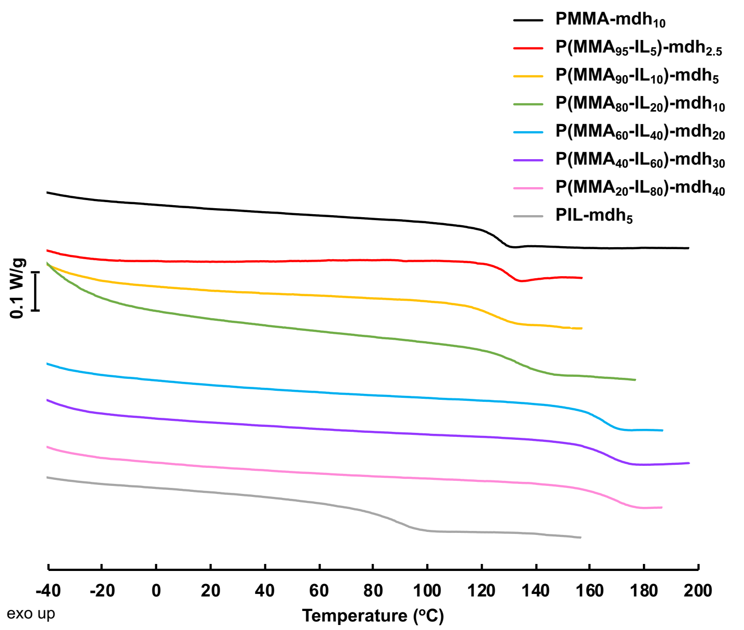

Figure S35. DSC second heating scans for **mdh**-crosslinked polymers.

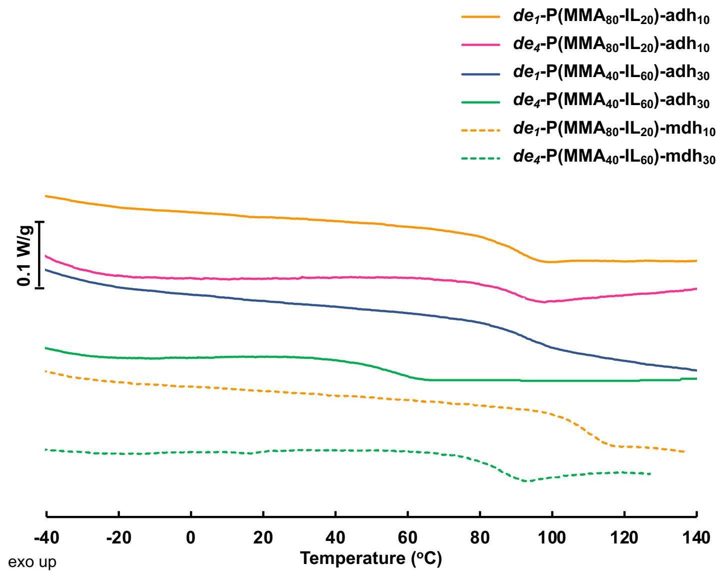

Figure S36. DSC second heating scans for de-crosslinked polymers.

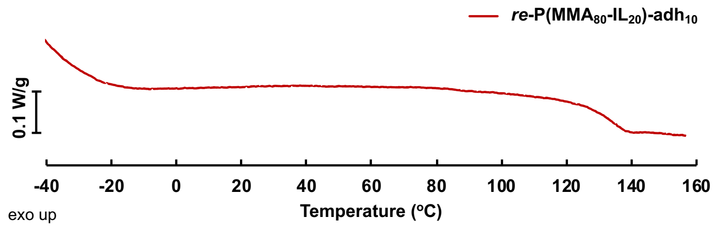

Figure S37. DSC second heating scan for re-crosslinked polymer.

7. SOLUBILITY

Table S1. Solubility of linear polymethacrylates, crosslinked polymers, de-crosslinked polymers, and a re-crosslinked polymer at 21 °C.

	solvent[a]								
polymer	H_2O δ = 48 (s)	CH_3OH δ = 30 (s)	*n*-BuOH δ = 23 (s)	DMSO δ = 25 (m)	THF δ = 19 (m)	Et_2O δ = 15 (m)	ACN δ = 24 (p)	$CHCl_3$ δ = 19 (p)	toluene δ = 18 (p)
PMMA	–	–	–	+	+	–	+	+	+
P(MMA_{95}-IL_5)	–	–	–	+	+	–	+	+	+
P(MMA_{90}-IL_{10})	–	–	–	+	+	–	+	+	+
P(MMA_{80}-IL_{20})	–	–	–	+	+	–	+	+	+
P(MMA_{60}-IL_{40})	–	–	–	+	+	–	+	+	–
P(MMA_{40}-IL_{60})	–	–	–	+	+	–	+	+	–
P(MMA_{20}-IL_{80})	–	–	–	+	+	–	+	+	–
PIL	–	–	–	+	+	–	+	+	–
PMMA-adh_{10}	–	–	–	+	+	–	+	+	+
P(MMA_{95}-IL_5)-$adh_{2.5}$	–	–	–	–	–	–	–	–	–
P(MMA_{90}-IL_{10})-adh_5	–	–	–	–	–	–	–	–	–
P(MMA_{80}-IL_{20})-$adh_{2.5}$	*n.d.*	*n.d.*	*n.d.*	*n.d.*	–	–	*n.d.*	–	*n.d.*
P(MMA_{80}-IL_{20})-adh_5	*n.d.*	*n.d.*	*n.d.*	*n.d.*	–	–	*n.d.*	–	*n.d.*
P(MMA_{80}-IL_{20})-adh_{10}	–	–	–	–	–	–	–	–	–
P(MMA_{60}-IL_{40})-adh_{20}	–	–	–	–	–	–	–	–	–
P(MMA_{40}-IL_{60})-adh_{30}	–	–	–	–	–	–	–	–	–
P(MMA_{20}-IL_{80})-adh_{40}	–	–	–	–	–	–	–	–	–
PIL-adh_5	–	–	–	–	–	–	–	–	–
PMMA-mdh_{10}	–	–	–	+	+	–	+	+	+
P(MMA_{95}-IL_5)-$mdh_{2.5}$	–	–	–	–	–	–	–	–	–
P(MMA_{90}-IL_{10})-mdh_5	–	–	–	–	–	–	–	–	–
P(MMA_{80}-IL_{20})-mdh_{10}	–	–	–	–	–	–	–	–	–
P(MMA_{60}-IL_{40})-mdh_{20}	–	–	–	–	–	–	–	–	–
P(MMA_{40}-IL_{60})-mdh_{30}	–	–	–	–	–	–	–	–	–
P(MMA_{20}-IL_{80})-mdh_{40}	–	–	–	–	–	–	–	–	–
PIL-mdh_5	–	–	–	–	–	–	–	–	–
de_1-P(MMA_{80}-IL_{20})-adh_{10}	*n.d.*	*n.d.*	*n.d.*	*n.d.*	+	–	*n.d.*	+	*n.d.*
de_4-P(MMA_{80}-IL_{20})-adh_{10}	*n.d.*	*n.d.*	*n.d.*	*n.d.*	+	–	*n.d.*	+	*n.d.*
de_1-P(MMA_{40}-IL_{60})-adh_{30}	*n.d.*	*n.d.*	*n.d.*	*n.d.*	+	–	*n.d.*	+	*n.d.*
de_4-P(MMA_{40}-IL_{60})-adh_{30}	*n.d.*	*n.d.*	*n.d.*	*n.d.*	+	–	*n.d.*	+	*n.d.*
de_1-P(MMA_{80}-IL_{20})-mdh_{10}	*n.d.*	*n.d.*	*n.d.*	*n.d.*	+	–	*n.d.*	+	*n.d.*
de_4-P(MMA_{40}-IL_{60})-mdh_{30}	*n.d.*	*n.d.*	*n.d.*	*n.d.*	+	–	*n.d.*	+	*n.d.*
re-P(MMA_{80}-IL_{20})-adh_{10}	*n.d.*	*n.d.*	*n.d.*	*n.d.*	–	–	*n.d.*	–	*n.d.*

[a]The symbols "+" and "–" indicate solubility and insolubility, respectively. Solubility parameters (δ, $MPa^{1/2}$) were obtained from the *Polymer Handbook* (J. Brandrup, E. H. Immergut, E. A. Grulke, A. Abe, D. Bloch. *Polymer Handbook, 4th ed.*, John Wiley and Sons, New York, 1999), and the letters s, m, and p denote strongly, moderately, and poorly hydrogen-bond-forming solvents, respectively. [b]*n.d.* – not determined.

REFERENCES

(1) Allaoua, I.; Goi, B. E.; Obadia, M. M.; Debuigne, A.; Detrembleur, C.; Drockenmuller, E. (Co)Polymerization of vinyl levulinate by cobalt-mediated radical polymerization and functionalization by ketoxime click chemistry. *Polym. Chem.* **2014**, *5* (8), 2973-2979.
